# Cetuximab plus capecitabine every three weeks as first-line maintenance therapy for RAS/BRAF wild-type metastatic colorectal cancer: a phase Ib dose-escalation study

**DOI:** 10.1038/s41698-026-01429-7

**Published:** 2026-04-24

**Authors:** Xiaoyu Xie, Ziqin Lin, Weiwei Li, Yuqian Xie, Jianwei Zhang, Huabin Hu, Shanshan Li, Xiaohui Zhai, Lishuo Shi, Yanhong Deng

**Affiliations:** 1 Department of Medical Oncology, The Sixth Affiliated Hospital, Sun Yat-sen University, Guangzhou, PR China; 2 Guangdong Provincial Key Laboratory of Colorectal and Pelvic Floor Diseases, Guangzhou, PR China; 3 Biomedical Innovation Center, The Sixth Affiliated Hospital, Sun Yat-sen University, Guangzhou, PR China; 4 State Key Laboratory of Oncology in South China, Guangzhou, PR China; 5 Clinical Research Center, The Sixth Affiliated Hospital, Sun Yat-sen University, Guangzhou, PR China

**Keywords:** Cancer, Gastroenterology, Oncology

## Abstract

Cetuximab plus fluoropyrimidine every two weeks (Q2W) is a common maintenance approach for RAS/BRAF wild-type metastatic colorectal cancer, but is limited by infusion burden and schedule misalignment. We conducted a phase Ib, nonrandomized 3 + 3 dose-escalation study evaluating cetuximab (400-700 mg/m^2^) every three weeks (Q3W) synchronized with capecitabine (1000 mg/m^2^ twice daily, days 1-14 every 21-day cycle) after first-line induction without disease progression. Six additional patients received standard cetuximab (500 mg/m^2^) plus infusional 5-fluorouracil/leucovorin every two weeks (Q2W) as a pharmacokinetic reference. Among 24 patients (18 every three weeks [Q3W]; 6 every two weeks [Q2W]), no maximum tolerated dose was reached up to 700 mg/m^2^ every three weeks (Q3W), and safety was consistent with known cetuximab and capecitabine-related toxicity. At 700 mg/m^2^ Q3W, cetuximab trough concentrations approached those observed with the Q2W reference regimen, and exploratory median progression-free survival was 13.4 months. These findings support cetuximab 700 mg/m^2^ Q3W plus capecitabine as the proposed recommended phase II dose (RP2D) regimen for further prospective evaluation. ClinicalTrials.gov: NCT05775900.

## Introduction

Colorectal cancer is the third most commonly diagnosed malignancy and the second leading cause of cancer-related mortality worldwide^1^. A substantial proportion of patients present with or eventually develop metastatic disease^2^, necessitating prolonged systemic therapy and maintenance strategies optimized for durability and convenience. Cetuximab, a chimeric IgG1 monoclonal antibody targeting the epidermal growth factor receptor (EGFR), inhibits signaling pathways that promote tumor cell proliferation, invasion, and metastasis^3^. In RAS/BRAF wild-type metastatic colorectal cancer (mCRC), particularly with left-side primary, cetuximab combined with fluoropyrimidine-based doublet chemotherapy constitutes a guideline-endorsed standard first-line approach supported by randomized clinical trials^4–10^. Following induction therapy, maintenance treatment is pivotal to prolong disease control while minimizing treatment burden^11,12^. Randomized studies have shown that maintenance therapy combining an anti-EGFR agent with a fluoropyrimidine backbone significantly improves progression-free survival (PFS) compared with either agent alone^13–15^. However, the fluoropyrimidine backbone in these studies often relies on continuous infusion of 5-fluorouracil (5-FU), which requires central venous access and pump support, and cumulative toxicities may reduce adherence. Therefore, oral fluoropyrimidine-based maintenance therapy represents an appealing alternative^16–18^. In a prospective phase II trial, reduced-dose capecitabine plus cetuximab achieved a median progression-free survival (mPFS) of 7.2 months and overall survival (OS) of 27.4 months, with manageable toxicity in patients with RAS/BRAF wild-type metastatic colorectal cancer (mCRC) following induction therapy^19^. Conference data have further supported the feasibility and potential benefit of cetuximab plus capecitabine maintenance therapy^20^. Despite encouraging outcomes, these regimens require weekly or every two weeks (Q2W) cetuximab infusions, which remain unsynchronized with the capecitabine schedule and impose logistical constraints. An every three weeks (Q3W) cetuximab regimen synchronizes with capecitabine’s 21-day treatment cycle and may reduce visit frequency and central venous access-related complications. Preliminary small-sample data suggest that Q3W cetuximab (750 mg/m^2^) is feasible and well tolerated^21^. Nonetheless, evidence regarding Q3W cetuximab regimens and prospective pharmacokinetic (PK) data in the maintenance setting remains limited. To address these limitations, we conducted a phase Ib dose-escalation study to evaluate the safety, pharmacokinetic (PK) profile, and exploratory efficacy of Q3W cetuximab plus capecitabine as first-line maintenance therapy in patients with RAS/BRAF wild-type metastatic colorectal cancer (mCRC).

## Results

### Patient characteristics

**Fig. 1:**
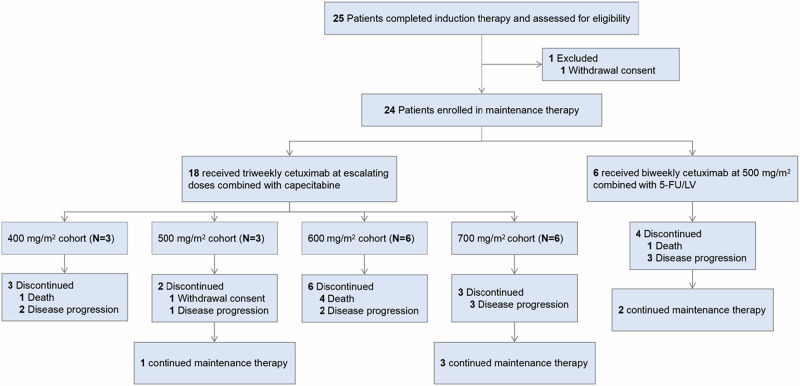
Trial flow diagram.

Between September 2022 and September 2024, a total of 25 patients were screened for eligibility and 24 patients were enrolled in the study. Of these, 18 patients received Q3W cetuximab plus capecitabine across four dose levels (400, 500, 600, and 700 mg/m^2^; *N* = 3, 3, 6, and 6, respectively), and 6 patients received the Q2W cetuximab regimen (Fig. 1). All enrolled patients received at least one treatment cycle and were included in the safety, efficacy, and pharmacokinetic analyses. No patients were lost to follow-up, and no major protocol deviations occurred. Baseline demographic and disease characteristics are summarized for all patients in Supplementary Table 1.

### Safety and dose-limiting toxicities (DLTs)

All 18 patients in the dose-escalation cohorts were evaluable for safety, and no unexpected toxicities were observed. No dose-limiting toxicities (DLTs) occurred among patients receiving cetuximab 400 mg/m^2^ or 500 mg/m^2^ Q3W in combination with capecitabine. After dose escalation, 1 of 6 patients treated at 600 mg/m^2^ experienced a dose-limiting toxicity (DLT), manifesting as grade 3 hand-foot syndrome and acneiform rash. Similarly, 1 of 6 patients treated at 700 mg/m^2^ developed a DLT, characterized by grade 3 hand-foot syndrome, acneiform rash, and skin fissures. The MTD was not reached. These grade 3 events were reversible and were managed with treatment interruption and supportive care. In both patients, treatment was delayed by one cycle, after which capecitabine was resumed at a reduced dose while cetuximab was maintained at the original dose. No further grade 3 toxicity was reported during subsequent treatment.

### Treatment-related adverse events (AEs)

All patients experienced at least one treatment-related adverse event (AE) during Q3W cetuximab plus capecitabine, with most events being grade 1 or 2. The most common treatment-related adverse events (AEs) were anemia (10/18, 56%), acneiform rash (10/18, 56%), transaminases increased (8/18, 44%), leukopenia (7/18, 39%), hand-foot syndrome (7/18, 39%), hyperbilirubinemia (5/18, 28%), neutropenia (4/18, 22%), mucosal inflammation (4/18, 22%) and diarrhoea (4/18, 22%) (Table 1). Two patients (2/18, 11%) experienced grade 3 treatment-related adverse events involving skin and subcutaneous tissue disorders (described in the DLTs section). No grade 4 or 5 treatment-related AEs were observed. No infusion-related reactions, cardiotoxicity, or treatment-related deaths occurred.

**Table 1:** Treatment-related adverse events (AEs)

MedDRA-preferred term, *n* (%)	Cetuximab Q3W										Cetuximab Q2W	
	400 mg/m^2^ (*N* = 3)		500 mg/m^2^ (*N* = 3)		600 mg/m^2^ (*N* = 6)		700 mg/m^2^ (*N* = 6)		All patients (*N* = 18)		500 mg/m^2^ (N = 6)	
	Grade 1/2	Grade 3/4	Grade 1/2	Grade 3/4	Grade 1/2	Grade 3/4	Grade 1/2	Grade 3/4	Grade 1/2	Grade 3/4	Grade 1/2	Grade 3/4
Any	3 (100)	-	3 (100)	-	6 (100)	1 (17)	6 (100)	1 (17)	18 (100)	2 (11)	6 (100)	1 (17)
Leukopenia	1 (33)	-	1 (33)	-	3 (50)	-	2 (33)	-	7 (39)	-	4 (67)	-
Neutropenia	-	-	-	-	3 (50)	-	1 (17)	-	4 (22)	-	3 (50)	-
Thrombocytopenia	-	-	-	-	2 (33)	-	-	-	2 (11)	-	1 (17)	-
Anemia	2 (67)	-	2 (67)	-	4 (67)	-	2 (33)	-	10 (56)	-	4 (67)	-
Hyperbilirubinemia	1 (33)	-	1 (33)	-	1 (17)	-	2 (33)	-	5 (28)	-	2 (33)	-
Hypokalaemia	1 (33)	-	1 (33)	-	-	-	-	-	2 (11)	-	-	-
Blood creatinine increased	-	-	-	-	1 (17)	-	-	-	1 (6)	-	1 (17)	-
Transaminases increased	1 (33)	-	2 (67)	-	3 (50)	-	2 (33)	-	8 (44)	-	2 (33)	-
Nausea	-	-	-	-	-	-	-	-	-	-	1 (17)	-
Vomiting	-	-	-	-	-	-	-	-	-	-	1 (17)	-
Decreased appetite	1 (33)	-	-	-	-	-	-	-	1 (6)	-	1 (17)	-
Fatigue	1 (33)	-	-	-	-	-	-	-	1 (6)	-	4 (67)	-
Mucosal inflammation	2 (67)	-	1 (33)	-	-	-	1 (17)	-	4 (22)	-	2 (33)	1 (17)
Skin fissures	-	-	-	-	-	-	-	1 (17)	-	1 (6)	-	-
Paronychia	1 (33)	-	-	-	-	-	-	-	1 (6)	-	2 (33)	-
Acneiform rash	2 (67)	-	1 (33)	-	2 (33)	1 (17)	3 (50)	1 (17)	8 (44)	2 (11)	6 (100)	-
Hand-foot syndrome	1 (33)	-	1 (33)	-	-	1 (17)	3 (50)	1 (17)	5 (28)	2 (11)	2 (33)	-
Abdominal pain	1 (33)	-	-	-	-	-	1 (17)	-	2 (11)	-	1 (17)	-
Diarrhoea	1 (33)	-	1 (33)	-	1 (17)	-	1 (17)	-	4 (22)	-	1 (17)	-
Abdominal distension	1 (33)	-	-	-	1 (17)	-	-	-	2 (11)	-	-	-
Constipation	1 (33)	-	1 (33)	-	1 (17)	-	-	-	3 (17)	-	1 (17)	-

### Pharmacokinetic analyses

**Fig. 2:**
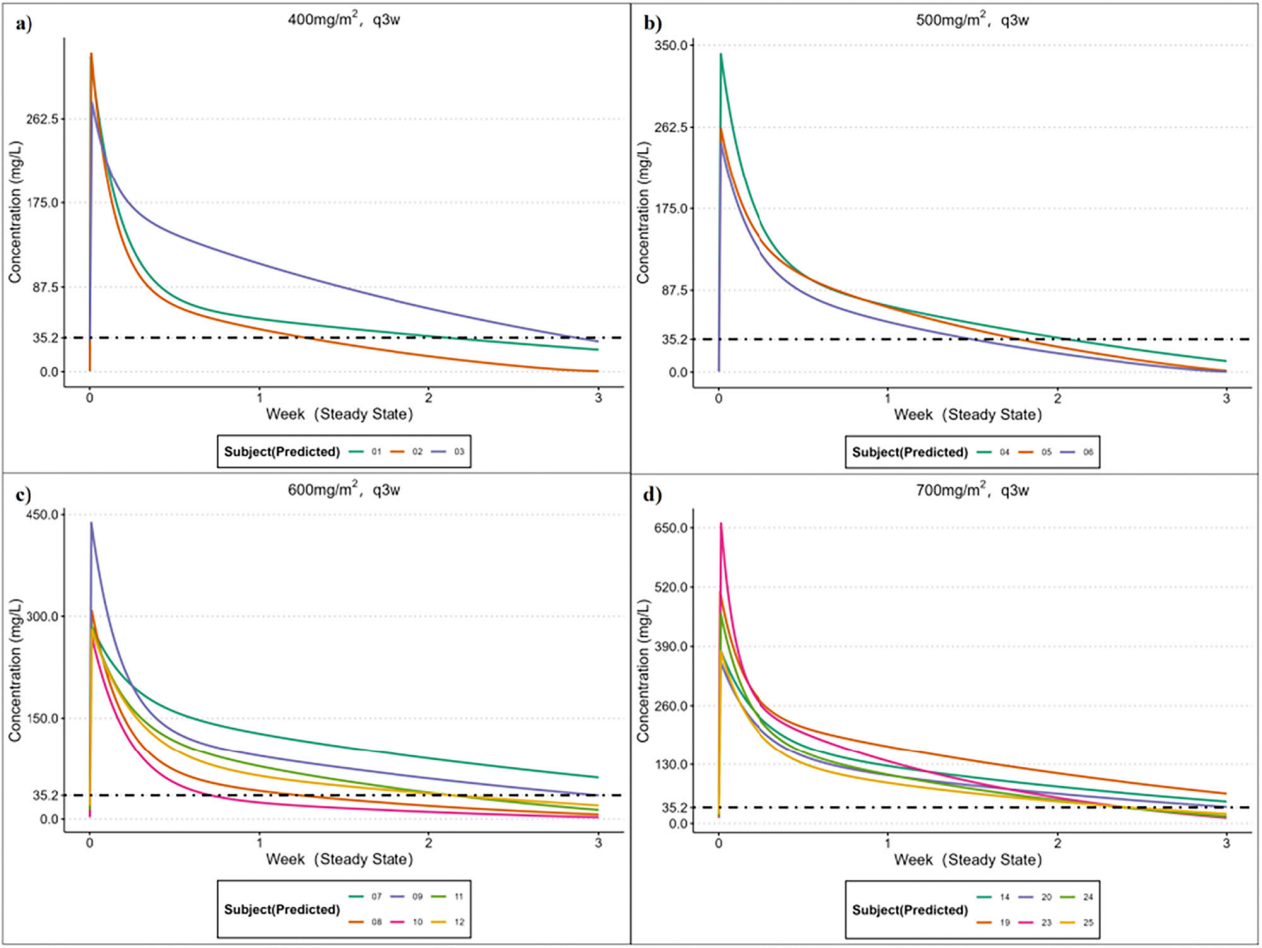
Individual steady state plasma concentration-time curves of cetuximab. **a** through **d** show the individual steady-state plasma concentration-time curves of cetuximab in patients receiving 400 mg/m^2^ Q3W (*N* = 3), 500 mg/m^2^ Q3W (*N* = 3), 600 mg/m^2^ Q3W (*N* = 6), and 700 mg/m^2^ Q3W (*N* = 6), respectively. In each panel, the black dashed line represents the mean trough concentration of 35.2 mg/L for the 500 mg/m^2^ Q2W dose group and served as the pharmacokinetic reference anchor. This anchor was used to evaluate whether each Q3W regimen achieved adequate pharmacologic coverage, as reflected by the duration of time during each dosing interval in which cetuximab concentrations remained above the reference line.

The population pharmacokinetic (popPK) model showed good agreement between predicted and observed values, and performed adequately in external validation (Supplementary Figs. 1 and 2). In addition to goodness-of-fit (GOF) and visual predictive check (VPC) analyses, the final popPK model showed acceptable precision for core structural parameters, and external predictive performance metrics supported overall model adequacy for exploratory PK bridging, although shrinkage was higher for some variability terms (Supplementary Tables 2, 3; and Supplementary Figs. 3, 4). Individual PK parameters estimated from the popPK model are summarized across all dosing groups in Supplementary Table 4. Following cetuximab plus capecitabine administration, serum concentration-time profiles exhibited a single peak, rising initially and then declining (Supplementary Fig. 5). The mean half-life of cetuximab was 131.7 ± 79.8 h in the 500 mg/m^2^ Q2W group, 125.1 ± 101.0 h in the 400 mg/m^2^ Q3W group, 31.7 ± 31.6 h in the 500 mg/m^2^ Q3W group, 144.8 ± 90.3 h in the 600 mg/m^2^ Q3W group, and 136.9 ± 70.4 h in the 700 mg/m^2^ Q3W group. Across the dose-escalation cohorts (400, 500, 600, and 700 mg/m^2^ Q3W), exposure generally increased with dose. Geomean values for area under the concentration-time curve (AUC_0-τ,ss_) and peak concentration (C_max,ss_) within the dosing interval at steady state increased with dose, whereas interpatient variability remained moderate. Trough concentrations (C_min,ss_) were markedly lower at 400, 500, and 600 mg/m^2^ Q3W compared with the 500 mg/m^2^ Q2W regimen. In contrast, the 700 mg/m^2^ Q3W cohort showed C_min,ss_ and AUC_0–τ,ss_ values numerically closer to those observed in the Q2W reference cohort than the lower Q3W dose levels, supporting pharmacokinetic bridging (Table 2). Based on simulated steady-state concentration profiles, the mean trough concentration of 35.2 mg/L from the 500 mg/m^2^ Q2W regimen served as the pharmacokinetic reference anchor. During a single 3-week dosing interval, the mean time below this reference anchor was 154.2 ± 134.4 h for the 400 mg/m^2^ Q3W group, 206.2 ± 47.2 h for the 500 mg/m^2^ Q3W group, 162.3 ± 155.4 h for the 600 mg/m^2^ Q3W group, and 49.7 ± 54.5 h for the 700 mg/m^2^ Q3W group (Fig. 2).

**Table 2:** Pharmacokinetic parameters of cetuximab at steady state

Pharmacokinetic parameters^a^		Cetuximab Q3W				Cetuximab Q2W
		400 mg/m^2^ (*N* = 3)	500 mg/m^2^ (*N* = 3)	600 mg/m^2^ (*N* = 6)	700 mg/m^2^ (*N* = 6)	500 mg/m^2^ (*N* = 6)
AUC_0-τ,ss_ (mg × h/L)	Mean (SD)	67,526.9 (26,858.9)	59,779.5 (10,164.8)	73,381.1 (31,175.4)	115,209.9 (24,433.6)	92,864.6 (38,509.6)
	Median	61,223.4	59,981.7	70,400.1	112,078.4	85,982.5
	Range	44,380.3, 96,976.9	49,515.2, 69,841.7	34,807.4, 119,577.3	87,750.7, 157,416.8	46,939.6, 145,946.0
	Geomean (CV%)	64,110.0 (39.8%)	59,195.8 (17.0%)	67,669.8 (42.5%)	113,188.9 (21.2%)	86,118.4 (41.5%)
C_max,ss_ (mg/L)	Mean (SD)	312.1 (28.4)	282.5 (50.9)	312.0 (63.0)	458.0 (114.9)	359.9 (80.1)
	Median	326.9	261.3	287.3	421.1	345.4
	Range	279.3, 330.2	245.7, 340.6	270.5, 438.0	355.6, 659.9	267.1, 497.0
	Geomean (CV%)	311.2 (9.1%)	279.6 (18.0%)	307.5 (20.2%)	447.2 (25.1%)	353.0 (22.2%)
C_min,ss_ (mg/L)	Mean (SD)	18.3 (15.8)	4.7 (6.2)	23.3 (21.9)	33.2 (20.9)	35.2 (30.9)
	Median	22.8	1.6	16.9	28.8	21.5
	Range	0.7, 31.3	0.6, 11.9	2.7, 61.6	12.4, 65.5	11.0, 82.6
	Geomean (CV%)	7.7 (86.7%)	2.3 (132.3%)	15.0 (93.9%)	27.8 (62.9%)	24.9 (87.8%)
C_av,ss_ (mg/L)	Mean (SD)	67.0 (26.6)	59.3 (10.1)	72.8 (30.9)	114.3 (24.2)	92.1 (38.2)
	Median	60.7	59.5	69.8	111.2	85.3
	Range	44.0, 96.2	49.1, 69.3	34.5, 118.6	87.1, 156.2	46.6, 144.8
	Geomean (CV%)	63.6 (39.8%)	58.7 (17.0%)	67.1 (42.5%)	112.3 (21.2%)	85.4 (41.5%)

^a^For the 500 mg/m^2^ Q2W dosing group, AUC_0-τ,ss_ was calculated as the sum of the areas under the concentration-time curve for three consecutive doses over a 6-week interval (*τ* = 6 weeks). C_max,ss_ and C_min,ss_ represent the mean peak and trough concentrations, respectively, across the three doses. For the dose-escalation Q3W groups, AUC_0-τ,ss_ was calculated over two consecutive doses within the same 6-week interval, with C_max,ss_ and C_min,ss_ reflecting the mean peak and trough concentrations from the two-dose interval. The average steady-state concentration (C_av,ss_) was derived as AUC_0-τ,ss_ divided by *τ* (6 weeks). *SD* = standard deviation, *CV* = Coefficient of Variation.

At 700 mg/m^2^ Q3W, the geometric mean trough concentration approached that observed in the 500 mg/m² Q2W cohort. The ratio of geomean for trough concentrations (C_min,ss_), area under the concentration-time curve (AUC_0-τ,ss_), and average steady-state concentration (C_av,ss_) between 700 mg/m^2^ Q3W and 500 mg/m^2^ Q2W regimens were 111.7, 131.4, and 131.4, respectively (Supplementary Table 5). Higher per-infusion doses at 700 mg/m^2^ Q3W led to increased peak concentration (C_max,ss_) values compared with Q2W dosing; however, these elevations did not translate into excess toxicity.

### Efficacy

As of the data cutoff on November 25, 2025, the median follow-up duration for all 24 patients was 26.1 months (95% CI, 17.8–34.4), and six deaths due to disease progression had occurred. Among the 18 patients treated with cetuximab plus capecitabine Q3W, best overall response was complete response in 2 patients (11%), partial response in 4 (22%), stable disease in 9 (50%), and progressive disease in 3 (17%). In exploratory analyses, maintenance median PFS was 9.7 months, 14.3 months, 8.6 months, and 13.4 months in the 400, 500, 600, and 700 mg/m^2^ Q3W cohorts, respectively (Table 3). Across all 18 patients treated with cetuximab plus capecitabine Q3W, the maintenance median PFS was 10.5 months. In the Q2W reference cohort, maintenance median PFS was 8.2 months. OS data remain immature because of the limited number of events.

**Table 3:** Efficacy

Efficacy variable	Cetuximab Q3W					Cetuximab Q2W
	400 mg/m^2^ (*N* = 3)	500 mg/m^2^ (*N* = 3)	600 mg/m^2^ (*N* = 6)	700 mg/m^2^ (*N* = 6)	All patients (*N* = 18)	500 mg/m^2^ (*N* = 6)
Response, *n* (%)						
Complete response	0 (0)	1 (33)	0 (0)	1 (17)	2 (11)	0
Partial response	0 (0)	0 (0)	2 (33)	2 (33)	4 (22)	1 (17)
Stable disease	2 (67)	2 (67)	2 (33)	3 (50)	9 (50)	3 (50)
Progressive disease	1 (33)	0 (0)	2 (33)	0 (0)	3 (17)	2 (33)
Overall response rate, %	0	33	33	50	33	17
Disease control rate, %	67	100	67	100	83	67
PFS, months^a^						
Median (95% CI)	9.7 (0, 22.8)	14.3 (9.7, 18.9)	8.6 (0, 18.0)	13.4 (NE, NE)	10.5 (9.1, 12.0)	8.2 (0, 18.5)

*NE* not evaluable.

^a^The median follow-up was 26.1 months (95% CI, 17.8–34.4).

## Discussion

For RAS/BRAF wild-type mCRC, maintenance therapy after induction with EGFR antibody-based doublet chemotherapy increasingly focuses on balancing durable disease control with cumulative toxicity and visit burden. Whereas bevacizumab plus capecitabine has long leveraged a Q3W rhythm in unselected or RAS/BRAF-mutant populations^17,18,22–24^, an analogous Q3W maintenance option within the anti-EGFR paradigm has been lacking. Previous studies have evaluated cetuximab administered Q3W at 750 mg/m^2^ during first-line induction therapy, effectively tripling the standard weekly dose of 250 mg/m^2^. However, simple arithmetic scaling does not ensure equivalent drug exposure, as monoclonal antibodies exhibit target-mediated drug disposition and schedule-sensitive trough variability. Our study addressed this gap by prospectively evaluating a Q3W cetuximab regimen synchronized with capecitabine’s 21-day cycle and by basing dose selection on PK comparability rather than empirical extrapolation. In this phase Ib study, no MTD was reached up to 700 mg/m^2^ Q3W. PK analyses showed that 700 mg/m^2^ Q3W provided pharmacokinetic bridging toward the steady-state C_min,ss_ and overall exposure (such as AUC_0-τ,ss_, C_av,ss_) of the 500 mg/m^2^ Q2W regimen, whereas lower Q3W dose levels (400–600 mg/m^2^) were associated with reduced trough and overall exposure. Safety was manageable, with most AEs limited to grade 1–2 and only 11% of patients experiencing grade 3 toxicities. The overall safety profile was consistent with the established toxicity spectrum of cetuximab and capecitabine in maintenance settings. Efficacy outcomes in the 700 mg/m^2^ Q3W group included an ORR of 50% and a DCR of 100%. At a median follow-up of 26.1 months, the mPFS was 13.4 months. These findings support 700 mg/m^2^ Q3W cetuximab plus capecitabine as a feasible PK-guided dose and schedule for further prospective evaluation.

Across the Q3W cohorts, exposure generally increased with dose, although some inconsistency was observed in the lower-dose groups. The unexpectedly lower PK parameters and the shorter half-life estimate in the 500 mg/m^2^ Q3W cohort should be interpreted cautiously, as they may reflect variability inherent to model-based estimation in very small cohorts. Notably, previously published cetuximab dose-escalation data using a Q2W schedule reported drug exposure increasing in an approximately dose-proportional manner and half-life estimates of approximately 133–156 h across 400–700 mg/m^2^ dose levels, broadly consistent with the half-life estimates observed in most cohorts of the present study and supporting cautious interpretation of the shorter half-life estimate and lower PK parameters in the 500 mg/m^2^ Q3W cohort as an isolated and unexplained observation^25^. Importantly, dose selection was based not on any single PK parameter, but on integrated steady-state exposure metrics (C_min,ss_, AUC_0-τ,ss_, and C_av,ss_), time below the Q2W reference trough anchor, and the observed safety profile. C_min,ss_ is the most clinically relevant anchor for maintenance because it correlates with sustained receptor occupancy and outcomes in prior exposure-response work^26–28^. Consistent with the PK rationale proposed in prior extended-interval cetuximab study^25^, we anchored dose selection to trough exposure and interdose pharmacologic coverage. Using the mean C_min,ss_ (35.2 mg/L) from the 500 mg/m^2^ Q2W group as a reference anchor, the time below threshold per three-week cycle was quantified, indicating that 700 mg/m^2^ Q3W provides more reliable pharmacologic coverage over the entire dosing interval (Fig. 2). This exposure comparability is further supported by the geomean ratio analysis: point estimates for C_min,ss_, C_av,ss_, and AUC_0-τ,ss_ with the 700 mg/m^2^ Q3W regimen were close to those of the 500 mg/m^2^ Q2W regimen, although the 90% CIs were wide and extended beyond conventional bioequivalence bounds. In contrast, the trough concentrations achieved with the 400–600 mg/m^2^ Q3W regimens were suboptimal and insufficient to ensure sustained therapeutic exposure.

One DLT at 600 mg/m^2^ coincided with a notably high peak concentration (C_max,ss_), while the DLT at 700 mg/m^2^ did not show abnormal C_max,ss_. This divergence suggests that toxicity is not tightly determined by peak concentration alone, and inter-individual susceptibility and cumulative exposure likely modulate risk. Importantly, despite higher per-infusion doses, the 700 mg/m^2^ Q3W cohort did not experience an excess or unexpected toxicity pattern; grade ≥3 AEs remained confined to dermatologic/hand-foot events in a small minority, mirroring historical cetuximab-capecitabine toxicities in maintenance contexts^19^. Toxicity differences between the Q3W and Q2W cohorts should not be attributed solely to cetuximab schedule, because the fluoropyrimidine backbone also differed between cohorts (oral capecitabine vs. infusional 5-FU/LV), which may have influenced the distribution of hand-foot syndrome, gastrointestinal toxicity, and mucosal toxicity. Notably, the two protocol-defined DLT events were reversible and manageable, and cetuximab could be continued at the original dose after recovery, suggesting that these toxicities should not be interpreted as evidence of Q3W schedule intolerance. Exploratory efficacy outcomes were numerically encouraging; however, these findings should be interpreted cautiously given the phase Ib design, small sample size, and potential baseline imbalances, including a higher proportion of left-sided primaries and lower multi-organ metastatic burden in the 700 mg/m^2^ cohort. However, CEA level and liver metastatic lesion count did not uniformly indicate the most favorable baseline tumor status in this cohort. In addition, survival outcomes may also have been influenced by subsequent management during maintenance, including local treatment in selected patients.

From a clinical standpoint, a Q3W cetuximab schedule offers several advantages. Synchronization with capecitabine’s 21-day cycle reduces hospital visits, infusion chair time, and healthcare costs. This convenience may improve adherence and quality of life, especially for elderly patients, those with comorbidities, or individuals living far from treatment centers. In an era emphasizing patient-centered care, these logistical benefits are increasingly important. Moreover, in resource-constrained settings, reducing infusion frequency without compromising efficacy has clear health-economic value.

This study has limitations. As a phase Ib, rule-based dose-escalation study, this trial was designed for dose selection and PK characterization rather than definitive efficacy evaluation or broad population-level inference. The absence of a randomized comparator precludes definitive efficacy conclusions, and outcomes may have been influenced by baseline imbalances. Pharmacokinetic analyses, though informative, were based on limited data. An additional limitation is that anti-drug antibodies (ADAs) were not assessed. Because ADAs may increase cetuximab clearance and reduce trough exposure, the lack of immunogenicity data may have contributed to PK variability. ADA assessment should therefore be included in future confirmatory studies. Given the phase Ib design, follow-up was relatively short, and overall survival data remain immature. These limitations highlight the need for validation in larger, controlled studies.

In summary, this phase Ib study provides early prospective PK evidence supporting a Q3W cetuximab schedule combined with capecitabine as maintenance therapy in RAS/BRAF wild-type mCRC. The 700 mg/m^2^ Q3W dose approached the exposure observed with standard Q2W dosing, was generally manageable from a safety perspective, and showed exploratory efficacy outcomes broadly consistent with prior maintenance studies. Accordingly, 700 mg/m^2^ Q3W is proposed as the recommended phase II dose (RP2D), based on integrated safety, PK exposure, and exploratory efficacy considerations. This patient-centered schedule warrants validation in larger randomized trials.

## Methods

### Study design and patient eligibility

This investigator-initiated, single-center, phase Ib dose-escalation trial was conducted at The Sixth Affiliated Hospital, Sun Yat-sen University (Guangzhou, China). The protocol was approved by the Ethics Committee, The Sixth Affiliated Hospital, Sun Yat-Sen University (Approval No. 2022ZSLYEC-632, December 27, 2022) and was conducted in accordance with the Declaration of Helsinki and Good Clinical Practice guidelines. The Ethics Committee also provided oversight of study safety during trial conduct. The study was registered at ClinicalTrials.gov (NCT05775900) on February 25, 2023. The trial protocol is available in the Supplementary Appendix.

Patients were consecutively recruited via investigator referral from the outpatient and inpatient clinics of the Department of Medical Oncology at The Sixth Affiliated Hospital, Sun Yat-sen University. All participants provided written informed consent prior to enrollment. Eligible participants were adults (≥18 years of age) with histologically confirmed RAS/BRAF wild-type mCRC. Participants must have completed first-line induction therapy with cetuximab plus FOLFOX or FOLFIRI and achieved disease control (complete or partial response, or stable disease) before initiation of maintenance therapy. Additional eligibility criteria included an Eastern Cooperative Oncology Group (ECOG) performance status (PS) of 0 or 1, a life expectancy of at least 12 weeks, and adequate bone marrow, hepatic, and renal function.

Dose escalation followed a standard 3 + 3 design with four planned Q3W dose levels (400, 500, 600, and 700 mg/m^2^), with an anticipated sample size of 3–24 patients (up to 6 per dose level). Eligible patients received cetuximab at escalating doses of 400, 500, 600, or 700 mg/m^2^ every three weeks (Q3W) combined with fixed-dose oral capecitabine (1000 mg/m^2^ twice daily on days 1–14 of each 21-day cycle). The evaluated Q3W dose levels were selected on the basis of toxicity considerations, the approved 500 mg/m^2^ Q2W cetuximab regimen, and the prior extended-interval cetuximab dose-escalation experience^25^. DLTs during the prespecified observation window were used to guide escalation and define the MTD. Dose escalation to the next cohort proceeded only after safety review. Upon completion of the DLT observation window in each cohort, the principal investigator and study team reviewed the safety data before escalation. To assess pharmacokinetic (PK) comparability with the standard Q2W cetuximab regimen (500 mg/m^2^ every 2 weeks [Q2W]), an additional six patients were enrolled. The recommended phase II dose (RP2D) was selected based on integrated evaluation of safety, pharmacokinetic exposure, and exploratory antitumor activity. No randomization or stratification was implemented. No blinding of participants, investigators, or outcome assessors was implemented.

### Procedures and investigational treatment

During first-line induction therapy, patients received cetuximab (500 mg/m^2^) every 2 weeks (Q2W), administered 1 h before either FOLFOX or FOLFIRI chemotherapy. Patients were enrolled after completing eight cycles of induction therapy and achieving disease control. Eligible patients received cetuximab (2-h infusion; administered intravenously under the supervision of the treating oncologist and trained oncology nurses in the infusion clinic of the Department of Medical Oncology at the Sixth Affiliated Hospital, Sun Yat-sen University) at escalating doses of 400, 500, 600, or 700 mg/m^2^ every 3 weeks (Q3W), combined with fixed-dose oral capecitabine (1000 mg/m^2^ twice daily on days 1–14 of each 21-day cycle). To enhance adherence, patients received medication counseling and were contacted by the treating physician via telephone after each treatment cycle. Treatment was continued in three-week cycles until disease progression, unacceptable toxicity, determination of the maximum tolerated dose (MTD), withdrawal of consent, or investigator decision. An additional six patients received the standard every two weeks (Q2W) regimen, which consisted of cetuximab (500 mg/m^2^; 2-h infusion), folinic acid (400 mg/m^2^ racemic or 200 mg/m^2^ L-form; 2-hour infusion), followed by an intravenous bolus of 5-fluorouracil (5-FU, 400 mg/m^2^) and a 46-h continuous infusion (2400 mg/m^2^). Treatment was continued in two-week cycles until disease progression, unacceptable toxicity, withdrawal of consent, or investigator decision.

### Outcomes

The primary endpoints were to evaluate the PK characteristics, assess safety, and determine the maximum tolerated dose (MTD) of cetuximab plus capecitabine every three weeks (Q3W). Secondary endpoints included progression-free survival (PFS), overall survival (OS), objective response rate (ORR), and disease control rate (DCR).

### Pharmacokinetic sampling and bioanalysis

Blood samples for cetuximab concentration measurement were collected within 1 h prior to cetuximab infusion (trough) and immediately after infusion (peak) at each scheduled cetuximab administration. Samples were processed to isolate serum and stored at −80 °C until analysis. Cetuximab concentrations were quantified using a validated ELISA.

### Population pharmacokinetic modeling and PK parameter derivation

The planned PK analysis of cetuximab injection is conducted based on data from a phase I trial (Protocol No. PROT-PS-17002, Version 1.0, October 9, 2018) and a phase III trial (Protocol No. PROT-PS-19001, Version 1.5, September 28, 2021) carried out by Guangdong ANNPO Biotechnology Inc., as well as on a population pharmacokinetic (popPK) model. A two-compartment population pharmacokinetic (popPK) model incorporating both linear and nonlinear (Michaelis-Menten) elimination pathways was used to characterize cetuximab disposition. This model incorporates covariates including body surface area, formulation, gender, study, albumin, and anti-drug antibody. The model includes a central compartment (Central) and a peripheral compartment (Peripheral). Linear clearance (CL) and intercompartmental clearance (*Q*) describe first-order elimination and distribution, respectively. Nonlinear elimination was modeled using Michaelis-Menten kinetics, defined by the maximum elimination rate (*V*_max_) and the Michaelis constant (*K*_m_), with C representing plasma drug concentration.

The equations representing the covariate effects are as follows:

1 $${\mathrm{CL}}={\theta }_{{CL}}\times {({BSA}/1.67)}^{1.57}\times (1.01\,{in\; Test\; Drug})$$

2 $${\rm{V}}1={\theta }_{V1}\times {({BSA}/1.67)}^{0.957}\times (0.944\,{in\; Test\; Drug})\times (0.871\,{in\; Female})\times (1.144\,{in\; Phase\; I})\times {(\mathrm{ALB}/41.53)}^{-0.333}$$

3 $${\rm{V}}2={\theta }_{V2}\times (0.833\,{in\; Test\; Drug})\times (0.834\,{in\; Female})$$

4 $${V}_{\max }={\theta }_{V\max }\times (0.895\,{in}\,{Test}\,{Drug})\,\times \,(0.846\,{in}\,{Female})\,\times \,(1.306\,{in}\,{ADA}\,{positive})$$

The study did not collect the covariate information of anti-drug antibody, and the samples were included in the model analysis with a default of ADA-negative results.

Model adequacy and external predictive performance were evaluated using goodness-of-fit plots (DV-PRED, DV-IPRED, CWRES-time, CWRES-PRED), visual predictive checks (1000 simulations), parameter precision assessed by relative standard errors (RSEs), shrinkage assessment for key interindividual variability and residual error terms, and summary predictive performance metrics in the external validation dataset. Observations with conditional weighted residuals outside ±6 were flagged as potential outliers and reviewed before exclusion. For the analysis dataset, missing concentration values and values below the lower limit of quantification were handled as missing dependent variables (MDV = 1), with corresponding values set to 0 and flagged; missing continuous covariates were imputed using the population (or subgroup) median and missing categorical covariates using the modal category.

Using the final model, steady-state concentration-time profiles were simulated over a 6-week dosing window for each regimen, corresponding to three administrations for Q2W dosing and two administrations for Q3W dosing. Steady-state PK parameters were derived from simulated profiles, including AUC_0-τ,ss_, C_max,ss_, and C_min,ss_ within the 6-week window; C_av,ss_ was calculated as AUC_0-τ,ss_/τ (6 weeks). PK comparability between the selected Q3W regimen and the reference Q2W regimen was assessed using geometric mean ratios with 90% confidence intervals.

### Safety and tumor assessments

Dose-limiting toxicity (DLT) was assessed during the first six weeks of treatment. DLT was defined as any grade 3 or 4 hematological or non-hematological toxicity considered treatment-related; treatment delays >14 days resulting in inability to receive planned dosing within the DLT window were also considered dose-limiting. The maximum tolerated dose (MTD) was defined as the highest dose level at which ≤1 of 6 patients experienced a DLT; dose escalation stopped if ≥2 patients at a dose level experienced DLTs. DLT classification was used for dose-escalation decision-making and did not necessarily mandate permanent treatment discontinuation; subsequent treatment interruption, dose delay, or treatment resumption was left to investigator discretion based on clinical recovery. Efficacy and safety assessments were conducted in the intention-to-treat and safety populations. Tumor response was evaluated by investigators according to Response Evaluation Criteria in Solid Tumors, Version 1.1 (RECIST 1.1). Baseline imaging of the chest, abdomen, and pelvis was performed using computed tomography (CT) or magnetic resonance imaging (MRI). Thereafter, radiologic assessments were conducted every nine weeks (every three Q3W cycles) to monitor disease progression. Treatment-emergent adverse events (AEs), occurring from the first day of study medication through 90 days after the final dose, were graded according to the National Cancer Institute Common Terminology Criteria for Adverse Events (NCI-CTCAE), Version 5.0.

Progression-free survival (PFS) was defined as the time from initiation of maintenance therapy to radiologically confirmed disease progression or death from any cause. Overall survival (OS) was defined as the time from initiation of maintenance therapy to death from any cause. Objective response rate (ORR) was defined as the proportion of patients achieving complete or partial response as assessed by investigators according to RECIST Version 1.1. Disease control rate (DCR) was defined as the proportion of patients who achieved complete response, partial response, or stable disease as their best overall response.

### Statistical analysis

The individual patient served as the unit of analysis for all safety, pharmacokinetic, and efficacy assessments. Descriptive statistics were used to summarize baseline characteristics, safety data, and pharmacokinetic parameters. Time-to-event endpoints were analyzed using the Kaplan–Meier method to estimate survival probabilities, with corresponding 95% confidence intervals calculated using Greenwood’s formula. Patients still alive at the time of analysis were censored for OS at the last known date alive. Patients without disease progression were censored for PFS at the date of their last valid radiological tumor assessment. The median follow-up time was estimated by the reverse Kaplan-Meier method. No formal hypothesis testing or subgroup analyses were planned because of the exploratory phase Ib design. Missing data were not imputed, and all analyses were based on available cases. Statistical analyses were performed using IBM SPSS Statistics (Version 22.0) and R (Version 4.3.0).

## Supplementary information

Supplement revised - 20260325 - clean

## Data Availability

The de-identified individual participant data that underlie the results reported in this article will be made available upon reasonable request to the corresponding author, subject to institutional approval and data sharing agreements. Please e-mail the corresponding author, Yanhong Deng at dengyanh@mail.sysu.edu.cn.

## Code availability

The code utilized in this research has not been publicly disclosed, however, it can be accessed by qualified researchers upon a reasonable request. Please e-mail the corresponding author, Yanhong Deng at dengyanh@mail.sysu.edu.cn
