# Supplementary material for: Cetuximab plus capecitabine every three weeks as first-line maintenance therapy for RAS/BRAF wild-type metastatic colorectal cancer: a phase Ib dose-escalation study
Source: NPJ Precis Oncol. 2026 Apr 24;10:250. doi: 10.1038/s41698-026-01429-7 (PMC13320180; doi:10.1038/s41698-026-01429-7)
Supplement: Supplementary file 1 — Supplement revised - 20260325 - clean [file 41698_2026_1429_MOESM1_ESM.pdf]

Contents

Supplementary Tables 2
Supplementary Table 1: Patient and disease characteristics at baseline 2
Supplementary Table 2: Final popPK pharmacokinetic model parameter estimates, RSEs, and shrinkage 4
Supplementary Table 3: External predictive performance metrics of model validation 5
Supplementary Table 4: Individual Pharmacokinetic (PK) parameters estimated from the population PK (popPK) model 6
Supplementary Table 5: Comparison PK parameters between the Q2W and Q3W dose groups 7
Supplementary Figures 8
Supplementary Fig. 1: Model goodness-of-fit (GOF) plot 8
Supplementary Fig. 2: Visual Predictive Check (VPC) plot of the population pharmacokinetic (popPK) model 9
Supplementary Fig. 3: Distributions of empirical Bayes estimates of inter-individual random effects 10
Supplementary Fig. 4: Distributions of prediction error metrics in external validation 11
Supplementary Fig. 5: Predicted steady-state plasma concentration-time profiles of cetuximab under different dosing regimens 12
Supplementary Appendix: Pharmacokinetic Analysis Protocol 13
Supplementary Appendix: Clinical Trial Protocol 23
Supplementary Appendix: CONSORT 2025 checklist 44

Supplementary Tables

Supplementary Table 1: Patient and disease characteristics at baseline

**Variable** *	**Cetuximab Q3W**					**Cetuximab Q2W**
	**400 mg/m^2 (N=3)**	**500 mg/m^2 (N=3)**	**600 mg/m^2 (N=6)**	**700 mg/m^2 (N=6)**	**All patients (N=18)**	**500 mg/m^2 (N=6)**
Age (years)						
Mean (SD)	59.3 (14.6)	56.3 (3.5)	56.3 (6.3)	60.0 (9.1)	58.1 (8.1)	60.7 (11.1)
Range	46-75	53-60	50-63	48-72	46-75	48-74
Gender, n (%)						
Male	2 (67)	3 (100)	5 (83)	4 (67)	14 (78)	3 (50)
Female	1 (33)	0 (0)	1 (17)	2 (33)	4 (22)	3 (50)
ECOG PS, n (%)						
0	1 (33)	3 (100)	5 (83)	4 (67)	13 (72)	3 (50)
1	2 (67)	0 (0)	1 (17)	2 (33)	5 (28)	3 (50)
Location of primary tumor, n (%)						
Left colon	1 (33)	1 (33)	3 (50)	6 (100)	11 (61)	4 (67)
Rectum	2 (67)	2 (67)	3 (50)	0 (0)	7 (39)	2 (33)
Differentiation, n (%)						
Well	0 (0)	0 (0)	0 (0)	0 (0)	0 (0)	0 (0)
Moderate	3 (100)	2 (67)	5 (83)	6 (100)	16 (89)	5 (83)
Poor	0 (0)	1 (33)	1 (17)	0 (0)	2 (11)	1 (17)
Time to metastasis, n (%)						
Synchronous	2 (67)	1 (33)	5 (83)	6 (100)	14 (78)	5 (83)
Metachronous	1 (33)	2 (67)	1 (17)	0 (0)	4 (22)	1 (17)
Number of metastatic sites, n (%)						
1	0 (0)	1 (33)	1 (17)	3 (50)	5 (28)	0 (0)

Variable *	Cetuximab Q3W					Cetuximab Q2W
	400 mg/m² (N=3)	500 mg/m² (N=3)	600 mg/m² (N=6)	700 mg/m² (N=6)	All patients (N=18)	500 mg/m² (N=6)
>1	3 (100)	2 (67)	5 (83)	3 (50)	13 (72)	6 (100)
Location of metastasis, n (%)						
Liver	3 (100)	2 (67)	3 (50)	5 (83)	13 (72)	4 (67)
Lung	2 (67)	1 (33)	3 (50)	1 (17)	7 (39)	2 (33)
Peritoneum	0 (0)	1 (33)	3 (50)	2 (33)	6 (33)	5 (83)
Supra-regional lymph nodes	3 (100)	1 (33)	1 (17)	2 (33)	7 (39)	4 (67)
Others	1 (33)	0 (0)	1 (17)	0 (0)	2 (11)	0 (0)
Number of liver metastases, n (%)						
≤3	3 (100)	2 (100)	2 (67)	1 (20)	8 (62)	1 (25)
>3	0 (0)	0 (0)	1 (33)	4 (80)	5 (38)	3 (75)
CEA , n (%)						
Normal (≤5ng/ml)	1 (33)	3 (100)	2 (33)	3 (50)	9 (50)	4 (67)
Abnormal (>5ng/ml)	2 (67)	0 (0)	4 (67)	3 (50)	9 (50)	2 (33)
Prior treatment, n (%)						
Cetuximab + FOLFOX	2 (67)	3 (100)	6 (100)	6 (100)	17 (94)	6 (100)
Cetuximab + FOLFIRI	1 (33)	0 (0)	0 (0)	0 (0)	1 (6)	0 (0)

* SD, standard deviation; ECOG PS, Eastern Cooperative Oncology Group (ECOG) performance status (PS).

Supplementary Table 2: Final popPK pharmacokinetic model parameter estimates, RSEs, and shrinkage

Parameters *	Final popPK pharmacokinetic model		
	Values	RSE (%)	Shrinkage (%)
Objective Function Value (OFV)	-8607.57		
Population parameter estimate			
CL (L/h)	0.0113	3.7%	
V1 (L)	2.54	1.8%	
Q (L/h)	0.0514	6.7%	
V2 (L)	3.37	3.8%	
V_{max} (L/h)	0.807	4.7%	
K_m (mg/L)	0.557	11.5%	
Interindividual variability			
IIV CL, OMEGA (%)	25.5%	3.9%	16.2%
IIV V1, OMEGA (%)	19.7%	3.1%	18.2%
IIV V2, OMEGA (%)	37.8%	3.2%	24.6%
IIV V_{max}, OMEGA (%)	16.3%	8.7%	56.2%
Residual error			
Additive (%)	16.6%	0.4%	16.9%

* RSE, relative standard error. Shrinkage is reported for interindividual variability terms and residual unexplained variability, where applicable.

Supplementary Table 3: External predictive performance metrics of model validation

Metrics *	Values
MPE	-0.16
MAPE	22.75
RMSE	39.07
MPE (%)	4.15%
MAPE (%)	18.12%
F20 (%)	72.81%
F30 (%)	83.13%

* PE, prediction error, PE = predicted value - observed value; MPE, median prediction error; MAPE, median absolute prediction error; RMSE, root mean square error; RPE: relative prediction error, RPE (%) = (predicted value - observed value) / observed value × 100%; MPE (%), mean relative prediction error; MAPE%, mean absolute relative prediction error; F20, percentage of RPE% within ±20%; F30, percentage of RPE% within ±30%.

Predictive performance metrics were calculated using individual model predictions and observed concentrations. These included prediction error (PE), mean prediction error (MPE), mean absolute prediction error (MAPE), root mean squared error (RMSE), relative prediction error (PE%), mean relative prediction error (MPE%), mean absolute relative prediction error (MAPE%), and the proportions of PE% within ±20% (F20) and ±30% (F30). In the present analysis, MPE, MAPE, RMSE, MPE%, MAPE%, F20, and F30 were -0.16, 22.75, 39.07, 4.15%, 18.12%, 72.81%, and 83.13%, respectively, indicating acceptable predictive performance of the model.

Supplementary Table 4: Individual Pharmacokinetic (PK) parameters estimated from the population PK (popPK) model

Pharmacokinetic parameters *		Cetuximab Q3W				Cetuximab Q2W
		**400 mg/m² (N=3)**	**500 mg/m² (N=3)**	**600 mg/m² (N=6)**	**700 mg/m² (N=6)**	**500 mg/m² (N=6)**
CL (L/h)	Mean (SD)	0.012 (0.000)	0.015 (0.001)	0.016 (0.008)	0.013 (0.003)	0.015 (0.006)
	Median	0.012	0.015	0.011	0.014	0.015
	Range	0.012, 0.012	0.013, 0.016	0.009, 0.029	0.008, 0.015	0.007, 0.023
	Geomean (CV%)	0.012 (1.896%)	0.015 (7.835%)	0.014 (50.631%)	0.012 (25.870%)	0.014 (40.978%)
V_{max} (L/h)	Mean (SD)	0.642 (0.100)	0.761 (0.121)	0.771 (0.121)	0.811 (0.057)	0.636 (0.106)
	Median	0.631	0.745	0.767	0.821	0.638
	Range	0.548, 0.748	0.649, 0.889	0.639, 0.982	0.719, 0.885	0.474, 0.782
	Geomean (CV%)	0.637 (15.630%)	0.755 (15.852%)	0.764 (15.623%)	0.809 (7.025%)	0.629 (16.645%)
V1 (L)	Mean (SD)	2.418 (0.222)	3.102 (0.253)	3.043 (0.297)	2.680 (0.523)	2.622 (0.620)
	Median	2.521	2.966	3.015	2.814	2.476
	Range	2.164, 2.571	2.947, 3.394	2.680, 3.403	1.797, 3.169	2.128, 3.831
	Geomean (CV%)	2.411 (9.185%)	3.096 (8.142%)	3.031 (9.773%)	2.632 (19.499%)	2.570 (23.637%)
V2 (L)	Mean (SD)	3.564 (0.289)	3.644 (1.320)	5.923 (2.324)	3.209 (0.918)	3.090 (0.892)
	Median	3.471	2.973	5.543	3.255	2.765
	Range	3.333, 3.888	2.793, 5.165	3.783, 9.942	1.803, 4.477	2.369, 4.739
	Geomean (CV%)	3.556 (8.114%)	3.501 (36.238%)	5.581 (39.231%)	3.088 (28.598%)	2.998 (28.870%)

* CL, the linear clearance from the central compartment; V_{max}, the maximum elimination rate via the nonlinear (Michaelis-Menten) pathway; V1, the volume of distribution in the central compartment; V2, the volume of distribution in the peripheral compartment; SD, standard deviation.

Supplementary Table 5: Comparison PK parameters between the Q2W and Q3W dose groups

**Pharmacokinetic parameters** *	**Geomean**			**Ratio of geomean**	**Ratio of geomean**
	**500mg/m² Q2W (N=6)**	**600mg/m² Q3W (N=6)**	**700mg/m² Q3W (N=6)**	**600mg/m² Q3W vs. 500mg/m² Q2W (90% CI)**	**700mg/m² Q3W vs. 500mg/m² Q2W (90% CI)**
$AUC_{0-\tau,ss}$ (mg*h/L)	86118.4	67669.8	113188.9	78.6 (49.5, 124.8)	131.4 (90.9, 190.0)
$C_{max,ss}$ (mg/L)	353.0	307.5	447.2	87.1 (70.8, 107.2)	126.7 (100.1, 160.3)
$C_{min,ss}$ (mg/L)	24.9	15.0	27.8	60.1 (20.4, 176.7)	111.7 (47.9, 260.8)
$C_{av,ss}$ (mg/L)	85.4	67.1	112.3	78.6 (49.5, 124.8)	131.4 (90.9, 190.0)

* For the 500 mg/m² Q2W dosing group, $AUC_{0-\tau,ss}$ was calculated as the sum of the areas under the concentration-time curve for three consecutive doses over a 6-week interval ($\tau = 6$ weeks).$C_{max,ss}$ and $C_{min,ss}$ represent the mean peak and trough concentrations, respectively, across the three doses. For the dose-escalation Q3W groups, $AUC_{0-\tau,ss}$ was calculated over two consecutive doses within the same 6-week interval, with $C_{max,ss}$ and $C_{min,ss}$ reflecting the mean peak and trough concentrations from the two-dose interval. The average steady-state concentration ($C_{av,ss}$) was derived as $AUC_{0-\tau,ss}$ divided by τ (6 weeks).

Supplementary Figures

Supplementary Fig. 1: Model goodness-of-fit (GOF) plot

Panels a) and b) show scatter plots of the observed cetuximab concentrations versus the population predicted concentrations and the individual predicted concentrations, respectively. Panel c) shows a scatter plot of the conditional weighted residuals (CWRES) versus the population predicted concentrations. Panel d) shows a scatter plot of the conditional weighted residuals versus time. In all panels, the blue circles represent the observed data points, and the red solid line represents the trend line. These goodness-of-fit plots indicate an overall acceptable agreement between the observed concentrations and both the individual and population model predictions, without an obvious time-dependent trend in the conditional weighted residuals.

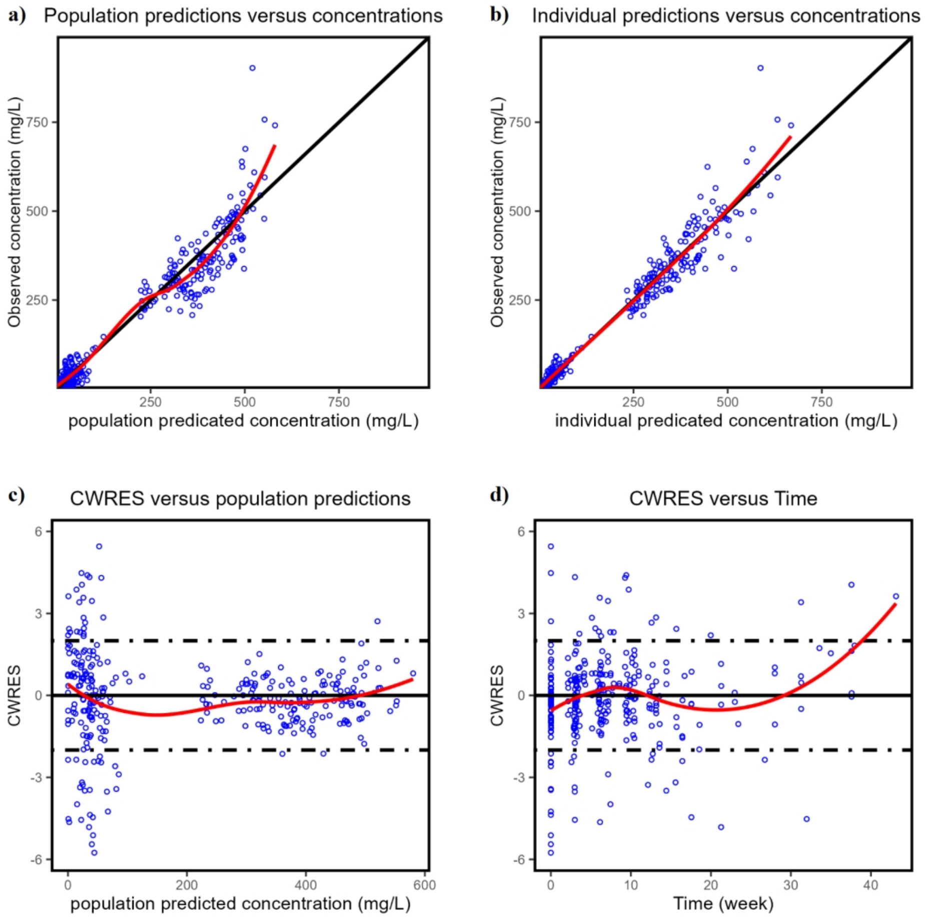

Supplementary Fig. 2: Visual Predictive Check (VPC) plot of the population pharmacokinetic (popPK) model

The blue hollow circles represent the observed cetuximab concentrations. The black dotted line, black solid line, and black dashed line represent the observed 10th percentile, 50th percentile, and 90th percentile, respectively. The corresponding model-simulated percentiles are shown by the red solid lines for the 10th percentile and 90th percentile and the blue solid line for the 50th percentile. The light red shaded areas and light blue shaded area indicate the 95% confidence intervals around the simulated percentile curves. The inset shows the early post-dose time period at an expanded scale. Overall, the observed percentile curves were generally contained within the 95% confidence intervals of the simulated percentiles, supporting an adequate predictive performance of the population pharmacokinetic model.

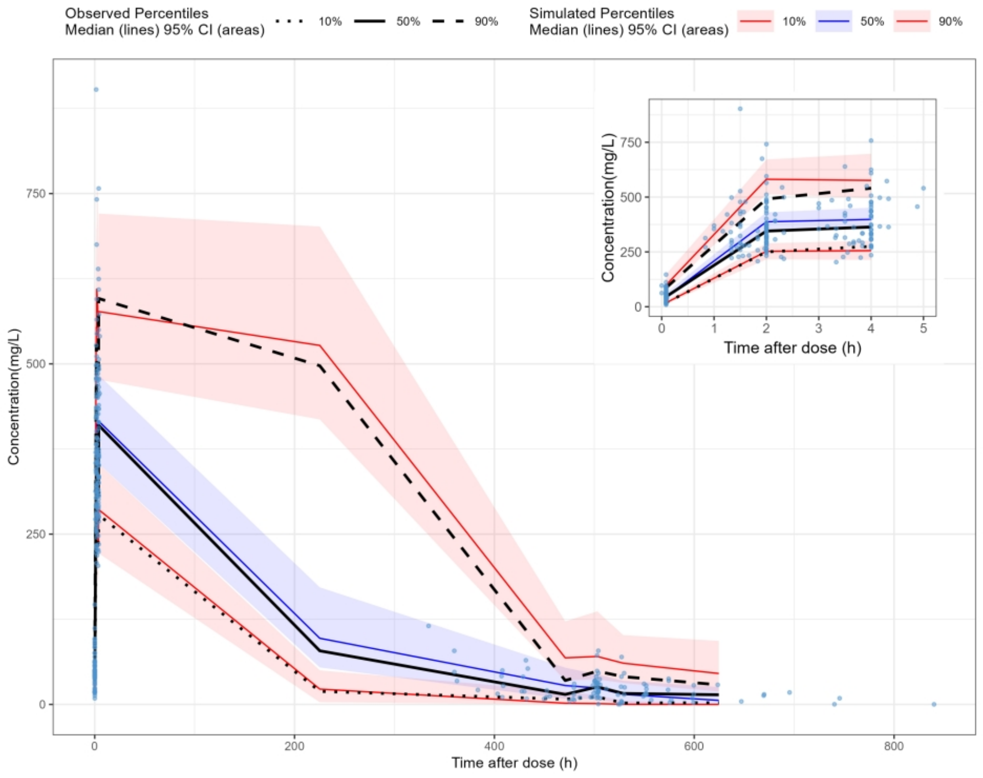

Supplementary Fig. 3: Distributions of empirical Bayes estimates of inter-individual random effects

Panels a) through d) show the histogram distributions of the empirical Bayes estimates of ETA for clearance (CL), central volume of distribution (V1), peripheral volume of distribution (V2), and maximum elimination rate (V_{max}), respectively, as measures of inter-individual variability in the population pharmacokinetic model. Distributions centered approximately around zero without marked skewness suggest the absence of obvious systematic bias in the corresponding random effects or prediction errors.

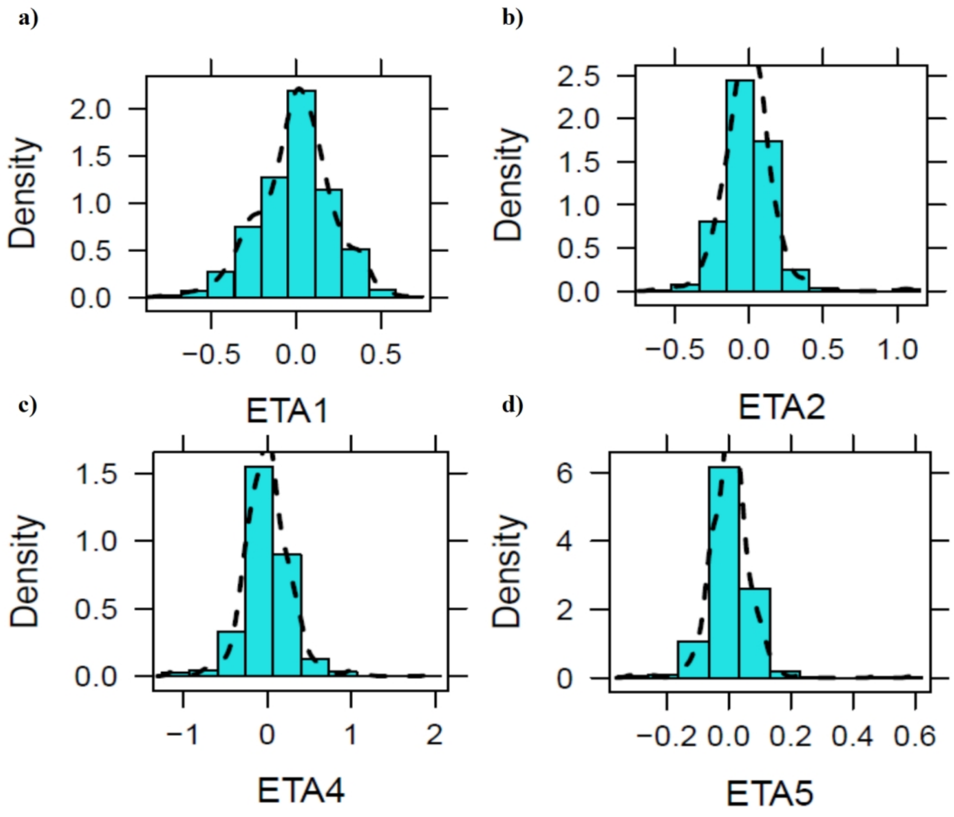

Supplementary Fig. 4: Distributions of prediction error metrics in external validation

Panels a) and b) show the histogram distributions of the external predictive performance metrics, including prediction error (PE) and relative prediction error (RPE%), respectively. PE and RPE% distributions centered near zero support limited overall prediction bias, whereas the spread of the distributions reflects the magnitude of prediction uncertainty.

PE, prediction error, PE = predicted value - observed value; RPE: relative prediction error, RPE (%) = (predicted value - observed value) / observed value × 100%.

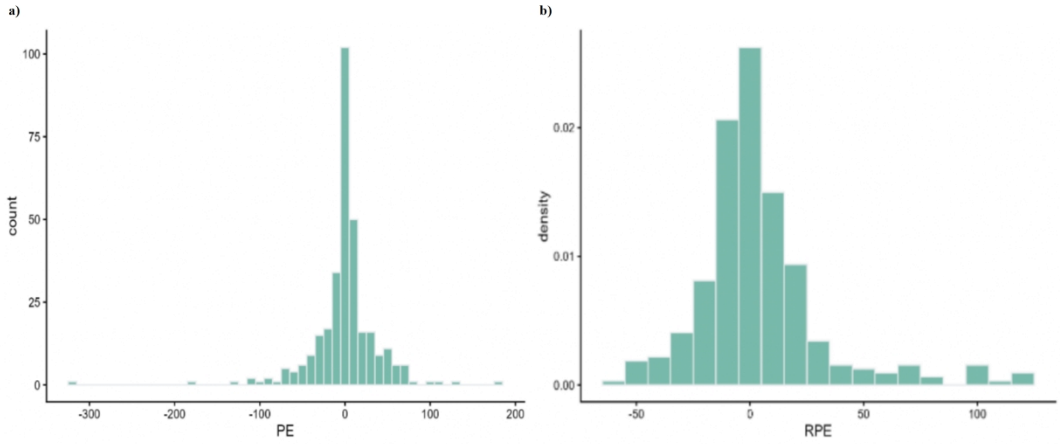

Supplementary Fig. 5: Predicted steady-state plasma concentration-time profiles of cetuximab under different dosing regimens

The curves show the model-predicted steady-state plasma concentration-time profiles of cetuximab for the 500 mg/m^2 every two weeks (Q2W) regimen and the 400 mg/m^2, 500 mg/m^2, 600 mg/m^2, and 700 mg/m^2 every three weeks (Q3W) regimens. Each curve represents the typical profile for one dosing group generated using the final population pharmacokinetic model. Across regimens, cetuximab concentrations rose rapidly after dosing to a single peak and then declined over time. This figure was used to visually compare the predicted exposure patterns among the different dosing regimens.

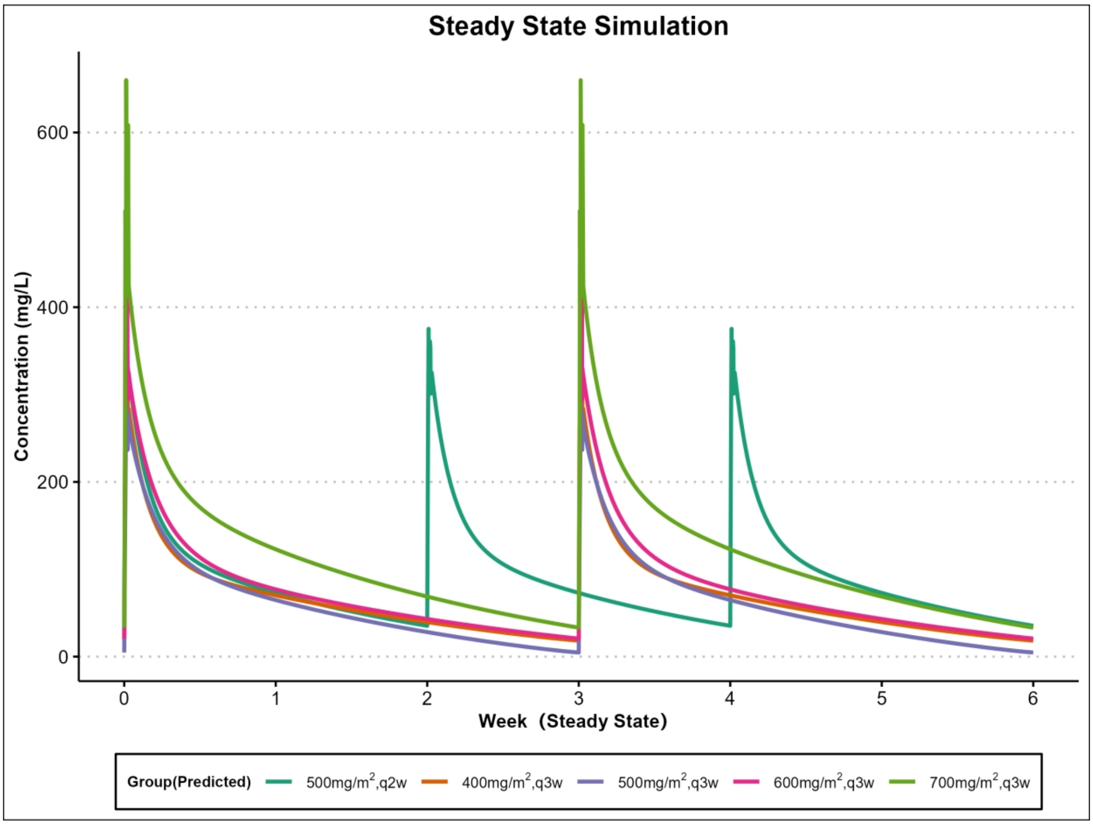

Supplementary Appendix: Pharmacokinetic Analysis Protocol

1. Introduction

Colorectal cancer (CRC) is the third most common malignancy and the second leading cause of cancer-related death worldwide. A substantial proportion of patients present with or eventually develop metastatic disease, requiring long-term systemic treatment and optimized maintenance strategies. Cetuximab, a chimeric IgG1 monoclonal antibody targeting the epidermal growth factor receptor (EGFR), inhibits signaling pathways that promote tumor proliferation, invasion, and metastasis. In RAS/BRAF wild-type metastatic colorectal cancer (mCRC), particularly left-sided tumors, cetuximab combined with fluoropyrimidine-based chemotherapy is a standard first-line regimen supported by clinical guidelines and randomized trials. Following induction therapy, maintenance treatment aims to sustain disease control while minimizing toxicity and treatment burden. Randomized studies have shown that anti-EGFR agents combined with a fluoropyrimidine backbone improve progression-free survival (PFS) over fluoropyrimidine monotherapy. However, panitumumab monotherapy failed to demonstrate non-inferiority to 5-FU/LV plus panitumumab, highlighting the importance of combination regimens. Continuous infusion of 5-FU requires central venous access and infusion pumps, contributing to logistical challenges and cumulative toxicities such as hematologic and gastrointestinal adverse events, which may reduce adherence and quality of life. Oral fluoropyrimidines like capecitabine offer a more convenient alternative. In a phase II study, reduced-dose capecitabine plus cetuximab yielded a median PFS of 7.2 months and OS of 27.4 months with manageable toxicity in RAS/BRAF wild-type metastatic colorectal cancer (mCRC). Conference data further suggested benefit for capecitabine plus cetuximab over cetuximab alone. However, prior studies administered cetuximab weekly or biweekly, which misaligns with capecitabine's 14-day-on, 7-day-off schedule. A every three weeks (Q3W) cetuximab regimen could synchronize with capecitabine's cycle, potentially reducing clinic visits, improving convenience, and minimizing infusion-related complications. While preliminary data suggest feasibility of every-three-weeks (Q3W) cetuximab at 750 mg/m², prospective pharmacokinetic (PK) data in the maintenance setting remain limited. Building on these observations, we conducted a phase Ib dose-escalation study to evaluate the safety, pharmacokinetics, and preliminary efficacy of every three weeks (Q3W) cetuximab in combination with capecitabine as first-line maintenance therapy in patients with RAS/BRAF wild-type mCRC.

According to the prescribing information of the originator cetuximab injection (Erbitux®), the recommended dosing regimens are as follows:

(1) An initial dose of 400 mg/m^2 followed by subsequent weekly doses of 250 mg/m^2;

(2) An initial and subsequent dose of 500 mg/m^2 administered every two weeks.

The planned pharmacokinetic (PK) analysis aims to assess the comparability of steady-state pharmacokinetic (PK) parameters between cetuximab administered every-three-weeks (Q3W) at doses of 400 mg/m^2, 500 mg/m^2, 600 mg/m^2, and 700 mg/m^2, and the label-recommended regimen of 500 mg/m^2 every two weeks.

This PK analysis of cetuximab injection (Erbitux®) is conducted based on data from a phase I trial (Protocol No. PROT-PS-17002, Version 1.0, October 9, 2018) and a phase III trial (Protocol No. PROT-PS-19001, Version 1.5, September 28, 2021) carried out by Guangdong ANNPO Biotechnology Inc., as well as on a population pharmacokinetic (popPK) model.

2. Study Objectives and data sources

Drawing on prior phase I and phase III clinical data of cetuximab together with a population pharmacokinetic (popPK) model, this planned PK analysis will evaluate the steady-state PK parameters

($C_{max, ss}$, $C_{min, ss}$, $AUC_{0-\tau, ss}$, etc.) across different dosing regimens, with a particular focus on assessing the comparability of steady-state PK profiles between the every-three-weeks (Q3W) and every-two-weeks (Q2W) administration schedules.

2.1 Clinical Trials

The PopPK model and source data were obtained from two prior studies conducted by Guangdong ANNPO Biotechnology Inc.: a phase I study in healthy volunteers (Protocol No. PROT-PS-17002, Version 1.0, October 9, 2018) and a phase III study in patients with metastatic colorectal cancer (Protocol No. PROT-PS-19001, Version 1.5, September 28, 2021). The designs of these two trials are summarized as follows.

2.1.1 Phase I Clinical Trial in Healthy Subjects (Protocol No. PROT-PS-17002)

Study Title:<br>A Single-Center, Randomized, Double-Blind, Phase I Comparative Clinical Trial Assessing the Safety, Pharmacokinetics, and Immunogenicity of Cetuximab Injection (APZ001) versus Erbitux® in Healthy Volunteers
Primary Objective:<br>A comparative assessment of the safety and pharmacokinetic profiles of cetuximab injection (APZ001) versus Erbitux® in healthy volunteers.
Study Design:<br>This pharmacokinetic study in healthy subjects was conducted as a randomized, double-blind, single-center clinical trial. Participants were assigned to one of two groups: the test group (T) received a single intravenous infusion of 250 mg/m^2 cetuximab injection (APZ001), and the reference group (R) received a single intravenous infusion of 250 mg/m^2 cetuximab injection (Erbitux®). Both treatments were administered over 2 hours, with antihistamines and corticosteroids given as premedication prior to infusion. Blood samples were collected at specified time points before and after dosing. Serum cetuximab concentrations were determined using a validated enzyme-linked immunosorbent assay (ELISA). Pharmacokinetic parameters, including AUC_{0-t}, C_{max}, $AUC_{0-\infty}$, T_{max}, and $t_{½}$, were derived and compared between the test and reference formulations to assess absorption rate, extent, and bioequivalence.
Study Drugs and Administration:<br>Test Formulation (T):<br>Cetuximab Injection; strength: 100 mg/20 mL per vial; manufacturer: Guangdong ANNPO Biotechnology Inc.; route of administration: intravenous infusion; dose: 250 mg/m^2; storage: 2-8 °C, protect from freezing. All doses administered to subjects will be from the same production batch.<br>A single intravenous infusion of 250 mg/m^2 cetuximab injection (APZ001) over 2 hours. Antihistamines and corticosteroids will be administered prior to infusion. Infusion time window: ±10 minutes.<br>Reference Formulation (R):<br>Cetuximab Injection (Erbitux®); strength: 100 mg/20 mL per vial; manufacturer: Merck KGaA, Germany; route of administration: intravenous infusion; dose: 250 mg/m^2; storage: 2-8 °C, protect from freezing. All doses administered to subjects will be from the same production batch.<br>A single intravenous infusion of 250 mg/m^2 cetuximab injection (Erbitux®) over 2 hours. Antihistamines and corticosteroids will be administered prior to infusion. Infusion time window: ±10 minutes.
Sample Size:

This study enrolled 100 healthy subjects in a staged manner. Participants randomly assigned to the test group (T, N=50) or the reference group (R, N=50).

PK Blood Sampling:

Approximately 4 mL of blood will be collected at the following time points: within 1 hour prior to dosing; at 1 hour during infusion; within 5 minutes after the end of infusion; and at 2.5, 3, 4, 6, 8, 24, 48, 96, 168, 264, 336, 432, 504, and 672 hours post-infusion initiation.

Samples will be collected into vacuum blood collection tubes and allowed to clot for at least 30 minutes, followed by centrifugation at 1200 g for 10 minutes at 4°C (acceptable temperature range: 2-8°C). The resulting serum will be aliquoted into labeled primary and backup cryovials and stored at ≤ -70°C until bioanalysis.

Analyte and Analytical Method for PK:

Pharmacokinetic samples will be analyzed using an enzyme-linked immunosorbent assay (ELISA).

2.1.2 Phase III Clinical Trial in Patients with Metastatic Colorectal Cancer (Protocol No. PROT-PS-19001)

Study Title:

A Multicenter, Randomized, Double-Blind, Parallel-Controlled Phase III Clinical Trial Evaluating the Efficacy, Safety, and Immunogenicity of Cetuximab Injection versus Erbitux®, Each in Combination with mFOLFOX6, as First-Line Therapy in Patients with RAS/BRAF Wild-Type Metastatic Colorectal Cancer

Primary Objective:

To evaluate and compare the efficacy of cetuximab injection and Erbitux®, each administered in combination with mFOLFOX6, as first-line therapy for patients with RAS/BRAF wild-type metastatic colorectal cancer, with the aim of demonstrating clinical equivalence.

Study Design:

This is a multicenter, randomized, double-blind, parallel-controlled phase III clinical trial designed to compare the efficacy, safety, and immunogenicity of cetuximab injection versus Erbitux®, each combined with mFOLFOX6, as first-line therapy in Chinese patients with RAS/BRAF wild-type metastatic colorectal cancer (mCRC). The primary objective is to determine whether cetuximab injection plus mFOLFOX6 is clinically equivalent to Erbitux® plus mFOLFOX6. The study will be conducted in three sequential phases: (1) a 24-week treatment period, (2) a continuation treatment period, and (3) a survival follow-up period.

Phase I: 24-week treatment period

Treatment cycles are defined as 2 weeks (14 days). Subjects without disease progression will continue cetuximab/Erbitux® plus mFOLFOX6 for 24 weeks or until one of the following occurs: lack of further clinical benefit, disease progression, unacceptable toxicity, investigator decision, withdrawal of informed consent, or death (whichever comes first). If intolerable toxicity attributable to cetuximab/Erbitux® occurs, chemotherapy may be continued. If chemotherapy is delayed or discontinued due to chemotherapy-related toxicity, cetuximab/Erbitux® treatment may continue. If toxicities related to both cetuximab/Erbitux® and chemotherapy occur, appropriate management and/or dose adjustment will be made in accordance with the protocol. No concomitant anti-cancer treatment is permitted during this phase. This phase will conclude 24 weeks after the first administration of the last enrolled subject. ORR data up to 24 weeks from the last subject will be analyzed and summarized in an interim report. Additional endpoints including PFS and OS will continue to be collected in patients entering Phase II.

Phase II: continuation treatment period<br>At the end of 24 weeks, subjects deemed by the investigator to be deriving ongoing clinical benefit and who consent to continue therapy will receive cetuximab injection (sponsor's product) until clinical benefit is no longer observed, disease progression, unacceptable toxicity, investigator decision, withdrawal of informed consent, or death. If additional anti-cancer therapies are initiated, the patient will proceed to the final tumor assessment visit. For evaluation of the primary endpoint ORR, imaging data and statistical analyses will undergo blinded review by an Independent Review Committee (IRC). Treatment decisions during the trial, however, will remain at the discretion of site investigators.<br>Phase III: survival follow-up period<br>Following treatment discontinuation, survival follow-up will be conducted every 3 months (±7 days) to obtain survival status and record subsequent anti-cancer therapies administered after the last dose. If dermatologic toxicities persist following the last administration, these adverse events will be monitored for up to 6 months post-treatment. Survival follow-up will continue for up to 36 months after randomization.
Study Drugs and Administration:<br>Experimental Arm (Cetuximab Injection):<br>An initial dose of 400 mg/m^2 will be administered as an intravenous infusion over 2 to 2.5 hours, at a maximum infusion rate of 5 mg/min (for patients with higher body weight, priority must be given to maintaining the infusion rate limit). Subsequent doses will be 250 mg/m^2 administered via intravenous infusion over at least 1 hour, at a maximum rate of 10 mg/min. Cetuximab will be given once weekly (every 7 days).<br>Control Arm (Erbitux®):<br>The method of administration is identical to that of the experimental arm.<br>mFOLFOX6 Regimen:<br>Oxaliplatin: 85 mg/m^2 on Day 1, intravenous infusion (minimum duration 2 hours).<br>Leucovorin (Calcium Folinate): 400 mg/m^2 on Day 1, intravenous infusion (minimum duration 2 hours).<br>5-Fluorouracil (5-FU): 400 mg/m^2 on Day 1, intravenous bolus injection (to be completed within 10 minutes).<br>5-Fluorouracil (5-FU): 2400 mg/m^2 continuous intravenous infusion over 46-48 hours.<br>This regimen will be repeated every 14 days (Q2W cycle).
Sample Size:<br>A total of 604 patients will be enrolled, with 302 assigned to the experimental arm and 302 to the control arm.
PK Blood Sampling:<br>Pharmacokinetic (PK) blood samples will be collected at designated time points before and after infusion of cetuximab or Erbitux®.<br>Sampling time points (relative to cetuximab/Erbitux® administration) are defined as follows: if dosing at a scheduled visit is delayed, blood sampling will be correspondingly postponed; if dosing is omitted at a visit, PK sampling will not be performed. The time window for pre-infusion sampling is within 60 minutes prior to dosing.<br>C1D1: pre-infusion; C1D1: within 5 minutes after end of infusion; C1D8: pre-infusion; C4D1: pre-infusion; C4D8: pre-infusion; C5D1: pre-infusion; C5D1: within 5 minutes after end of infusion; C5D8: pre-infusion.

At each time point, 4 mL of whole blood will be drawn into a vacuum blood collection tube, allowed to clot for 30-60 minutes, and centrifuged at 1200-1700 g for 10 minutes. The resulting serum will be aliquoted into two pre-labeled cryovials and stored at -70 °C in an ultra-low temperature freezer (-20 °C permitted for temporary storage).
Analyte and Analytical Method for PK:<br>Serum concentrations of cetuximab will be determined by a validated enzyme-linked immunosorbent assay (ELISA).

2.2 Analysis Dataset

2.2.1 Software

Analysis datasets compatible with NONMEM will be created using R (version 4.3.0) or Microsoft Excel and saved in .CSV format.

2.2.2. Data Inclusion Criteria

The analysis will include NONMEM PK datasets generated from previous studies (PROT-PS-17002 and PROT-PS-19001) and all subjects who received cetuximab injection and had at least one valid post-dose serum concentration measurement in the phase Ib dose-escalation study.

2.3 Covariates

Covariates will be categorized as fixed or time-varying. Fixed covariates are variables that remain constant during the study period (e.g., sex), whereas time-varying covariates may change during the study (e.g., laboratory parameters).

Potential covariates that may influence cetuximab pharmacokinetics include:

(1) Demographic variables: age (years), sex, body weight (kg), body surface area (m^2).

(2) Age categories: 0 = <40 years; 1 = 40-<65 years; 2 = ≥65 years.

(3) Clinical laboratory variables: serum albumin, white blood cell count, etc.

(4) Disease-related variables: performance status, etc.

2.4 Handling of Missing Data

Dosing data: Subjects with missing dosing information (dose or time) will be reviewed individually to determine eligibility for inclusion in the analysis.

PK data: Missing drug concentration values will be assigned as 0, with the corresponding MDV (missing dependent variable) coded as 1. Values below the lower limit of quantification (LLOQ) will also be set to 0, with MDV coded as 1 and an LLOQ flag applied.

Covariates: Missing continuous covariates will be imputed using the median value of the overall population or corresponding subgroup. Missing categorical covariates will be imputed using the most frequent category within the dataset.

3. Pharmacokinetic Analysis

3.1 Overall Approach

Based on the data from previous studies (PROT-PS-17002 and PROT-PS-19001) and the population pharmacokinetic (popPK) model, this model is a two-compartment model with mixed linear and nonlinear Michaelis-Menten elimination, which incorporates covariates including body surface area (BSA), formulation (GRP), gender (GENDER), study (SID), albumin (ALB), and anti-drug antibody (ADA).

The schematic diagram of the structural model is as follows:

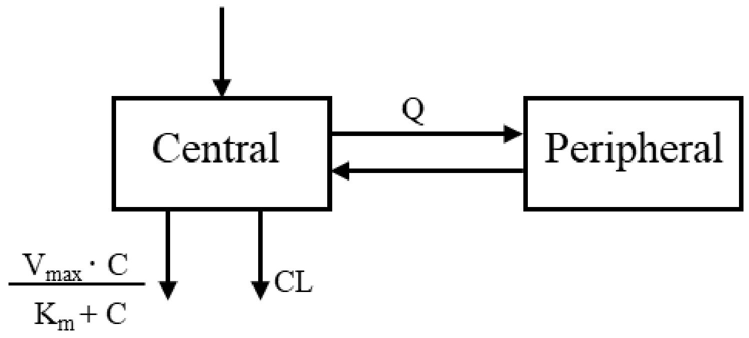

Figure 1. Schematic Diagram of the PK Structural Model

A two-compartment population pharmacokinetic (popPK) model incorporating both linear and nonlinear (Michaelis-Menten) elimination pathways was used to characterize cetuximab disposition. The model includes a central compartment (Central) and a peripheral compartment (Peripheral). Linear clearance (CL) and intercompartmental clearance (Q) describe first-order elimination and distribution, respectively. Nonlinear elimination was modeled using Michaelis-Menten kinetics, defined by the maximum elimination rate (V_{max}) and the Michaelis constant (K_m), with C representing plasma drug concentration.

The equations representing the covariate effects are as follows:

(1) $CL = \theta_{CL} \times (BSA/1.67)^{1.57} \times (1.01\ \textit{in Test Drug})$

(2) $V1 = \theta_{V1} \times (BSA/1.67)^{0.957} \times (0.944\ \textit{in Test Drug}) \times (0.871\ \textit{in Female}) \times (1.144\ \text{in Phase I}) \times (ALB/41.53)^{-0.333}$

(3) $V2 = \theta_{V2} \times (0.833\ \textit{in Test Drug}) \times (0.834\ \textit{in Female})$

(4) $V_{max} = \theta_{Vmax} \times (0.895\ \textit{in Test Drug}) \times (0.846\ \textit{in Female}) \times (1.306\ \textit{in ADA positive})$

Note: The exploratory study did not collect the covariate information of anti-drug antibody (ADA), and the samples were included in the model analysis with a default of ADA-negative results.

External validation of the popPK model was performed using exploratory PK data:

(1) If the external validation is passed, the popPK model from previous studies will be used for concentration simulation, and then the steady-state PK parameters will be calculated;

(2) If the external validation fails, the data of Erbitux® (the innovator drug of cetuximab) from previous studies (PROT-PS-17002 and PROT-PS-19001) and the phase Ib dose-escalation study PK data will be integrated to reconstruct the popPK model. Concentration simulation will be conducted based on the newly constructed popPK model, and then the steady-state PK parameters will be calculated.

3.2 Software and Hardware

NONMEM (Version 7.5.0 or higher) was used for nonlinear mixed-effects modeling and simulation. Pirana (Version 2.9 or higher) and PsN were used to run NONMEM. If necessary, Excel, WinNonlin, R, or SAS could be adopted for tasks such as generating diagnostic plots and conducting result processing. Any software used in the analysis and its corresponding version will be documented in the summary report.

3.3 External Validation of the Model

Goodness of Fit (GOF) plots were generated, including the scatter plot of dependent variable vs. population predicted value (DV-PRED), scatter plot of dependent variable vs. individual predicted value (DV-IPRED), scatter plot of Conditional Weighted Residuals vs. time (CWRES-TIME), and scatter plot of Conditional Weighted Residuals vs. predicted value (CWRES-PRED). Data points with Conditional Weighted Residuals (CWRES) outside the range of ±6 were regarded as outliers, and after verification, a decision would be made on whether to exclude them.

The Visual Prediction Check (VPC) method was used, which is a model simulation-based verification method. Graphs were plotted to compare the distribution characteristics of simulated data and observed data. Based on the previous popPK model, 1000 simulations were performed on the exploratory PK data. If the median, 5th percentile, and 95th percentile of the observed data in the graph were basically within the 95% confidence interval of the corresponding percentiles of the predicted data, it was considered that the previous popPK model had a good overall predictive performance for the PK exploratory study data, and the external validation was passed.

3.4 Reconstruction of the Model

Integrate the data of Erbitux® (the innovator drug of cetuximab) from previous studies (PROT-PS-17002 and PROT-PS-19001) and the phase Ib dose-escalation study PK data to establish a population pharmacokinetic (popPK) model for Erbitux® (the innovator drug of cetuximab). A base model capable of describing the plasma concentration of cetuximab was selected. Starting with the one-compartment model, linear elimination, nonlinear elimination, or parallel linear and nonlinear elimination pathways were tested. Initially, all structural parameters included between-subject variability (IIV); however, to ensure successful model convergence, it may be necessary to reduce the number of between-subject variability parameters. Relevant structural parameters may also include inter-occasion variability (IOV). After the base model is determined, the influence of covariates is then evaluated.

3.4.1 Exploratory Data Analysis

Summary statistics were calculated to describe the number and characteristics of subjects to be modeled in the total dataset. Plots were generated including: frequency distribution plots of subject characteristics, box plots of continuous characteristics relative to distribution features, scatter plots of the relationships between continuous characteristics (if there is a correlation between covariates ($r \geq 0.7$), one representative covariate was selected for subsequent investigation), and concentration-time curves.

3.4.2 Identification and Handling of Outliers

Before modeling, outliers that significantly deviate from the population distribution were identified by the graphical method, and a decision on whether to exclude them was made after verification. During modeling, data points with Conditional Weighted Residuals (CWRES) outside the range of ±6 were regarded as outliers, and a decision on whether to exclude them was made after verification. All excluded data will be listed in the report.

3.4.3 Estimation Methods

First-Order Conditional Estimation with Interaction (FOCEI) served as the initial method for parameter estimation. If FOCEI fails to converge to reliable parameter estimates, alternative methods such as the Monte Carlo Importance Sampling Expectation-Maximization Algorithm (IMP) and Stochastic Approximation Expectation-Maximization (SAEM) could be adopted. When using the IMP and SAEM methods, 'Mu Reference' was employed to improve computational efficiency.

3.4.4 Investigation of the Basic Structure

For the population pharmacokinetic (popPK) model, different PK structural models were investigated and compared (e.g., one-compartment model, two-compartment model, linear clearance model, nonlinear clearance model, parallel linear and nonlinear clearance models, or other appropriate models investigated based on data characteristics, etc.).

3.4.5 Investigation of Statistical Models

The random effects model includes between-subject variability (BSV) and residual variability (RV).

Between-subject variability refers to the random error among study subjects. The exponential model

was adopted for the between-subject variability model.

$$P_i = P_{TV} \times \exp(\eta_i)$$

In the above equation, P_{TV} refers to the population typical value of the parameter, P_i denotes the individual parameter value, and η_i represents the between-subject random effect, which follows a normal distribution with a mean of 0 and a variance of ω^2.

For residual variability (RV), the following models were investigated respectively: additive model, proportional model, and combined model.

$$Y_{obs,ij} = Y_{pred,ij} + \varepsilon_{ij,1}$$

$$Y_{obs,ij} = Y_{pred,ij} \times (1 + \varepsilon_{ij,2})$$

$$Y_{obs,ij} = Y_{pred,ij} \times \left(1 + \varepsilon_{ij,2}\right) + \varepsilon_{ij,1}$$

In the above context, $Y_{obs,ij}$ and $Y_{pred,ij}$ refer to the observed value and predicted value of the effect, respectively; $\varepsilon_{ij,1}$ represents the additive within-subject random effect (variability), and $\varepsilon_{ij,2}$ represents the proportional within-subject random effect (variability). $\varepsilon_{ij,1}$ and $\varepsilon_{ij,2}$ follow a normal distribution with a mean of 0 and variances of $\sigma_{ij,1}^2$ and $\sigma_{ij,2}^2$ respectively.

Based on the Objective Function Value (OFV), goodness-of-fit (GOF) plots, as well as the rationality and stability of parameters, the fitting results of different error models were compared respectively to select the optimal model.

3.4.6 Covariate Analysis

A set of population pharmacokinetic (popPK) parameters that minimize the Objective Function Value (OFV) was identified. The OFV among models of the same type approximately follows a chi-square (χ^2) distribution: when the degrees of freedom (df) = 1, $\chi^2_{0.01,1}$ = 6.64 and $\chi^2_{0.001,1}$ = 10.83. That is, when the number of parameters differs by 1 between two models, if ΔOFV > 6.64, there is a significant difference with P < 0.01; if ΔOFV > 10.83, there is a significant difference with P < 0.001.

The effect of continuous variables was incorporated into the model in the form of a power function.

$$P_{TV} = \theta_{TV} \times \left(\frac{COV_i}{median}\right)^{\theta_1}$$

Among them, P_{TV} refers to the population parameter value of the parameter, θ_{TV} is the estimated value of the parameter when the individual covariate is equal to the covariate median, COV_i and median represent the covariate and its median respectively, and θ_1 represents the degree of influence of the covariate on the parameter.

Discrete variables need to be categorized before being incorporated into the model, and the IF format is used to incorporate covariates.

$$\text{IF}(COV_i\text{.EQ.x})P_{TV} = \theta_{TV} \times \theta_1$$

Among them, P_{TV} refers to the population parameter value of the parameter, θ_{TV} is the estimated value of the parameter when the individual covariate is equal to the default value of the covariate (reference group), COV_i and x represent the covariate and the value of the non-reference group respectively, and θ_1 represents the degree of influence of the covariate on the parameter.

After the base model is determined, the effect of body surface area (BSA) on PK parameters is first incorporated, and then other covariates are screened. The covariates with potential influence are identified based on the correlation between the parameter ETA values and covariates.

3.4.6.1 Forward Selection Process

First, forward covariate modeling is performed to obtain the full model. For the established base model, each pre-selected covariate that may have an impact is tested separately, and the significance of the covariate is determined through the Likelihood Ratio Test (LRT). The LRT judges whether a covariate

has statistical significance based on the number of additional parameters before and after adding the covariate and the change in the Objective Function Value (OFV). When the degree of freedom (df) is 1, an OFV change greater than 6.64 corresponds to $p < 0.01$. Therefore, in this study, when a covariate is added to the expression of a parameter, if the reduction in the OFV is more than 6.64, the covariate is considered to have a significant impact on the parameter; covariates that cause a significant decrease in the model's OFV and have reasonable parameter fitting values are regarded as meaningful covariates for further screening. Among these, the covariate with the smallest p-value and the most significant impact on the parameter is selected and added to the model, which serves as the base model for this step. Then, all remaining covariates that have significant meaning for the parameter are tested, and the covariate with the smallest p-value among them is added to the model. The above steps are repeated until no new covariates have a significant impact on the parameter, and the full model (Full Model) is obtained.

3.4.6.2 Backward Elimination Process

After obtaining the full model, backward modeling is conducted to get the final model. Specifically, one covariate is sequentially eliminated from the full model while the remaining covariates are retained, and the Likelihood Ratio Test (LRT) is used to determine whether the covariate has statistical significance at the $p < 0.001$ level. If the increase in the Objective Function Value (OFV) after eliminating a covariate exceeds the criterion (ΔOFV > 10.83), the covariate is retained in the model. This step is repeated until all covariates meet the set criteria and the parameter fitting values are reasonable (excluding those with Relative Standard Error (RSE) > 35%), and the resulting model is the final model.

3.4.7 Model Evaluation

A combination of diagnostic plots and statistical tests was used for model evaluation.

Goodness of Fit (GOF) plots were generated, including scatter plots of dependent variable vs. population predicted value (DV-PRED), dependent variable vs. individual predicted value (DV-IPRED), Conditional Weighted Residuals vs. time (CWRES-TIME), Conditional Weighted Residuals vs. predicted value (CWRES-PRED), scatter plots of parameters and between-subject variability, and scatter plots of the correlation between between-subject variability and covariates. These plots aimed to compare the consistency between observed and predicted values, the distribution and trend of residuals, and the correlation between various parameters to guide model optimization.

The Visual Prediction Check (VPC) is a model simulation-based verification method that involves plotting graphs to compare the distribution characteristics of simulated data and observed data. In this study, 1000 simulations were performed. If the median, 5th percentile, and 95th percentile of the observed data in the graph fell within the 95% confidence interval of the corresponding percentiles of the predicted data, the distribution characteristics were considered similar, and the model had good overall predictive performance.

The Bootstrap method is a resampling method with replacement. It calculates statistics and estimates the sample distribution using sample data without making any assumptions about the model. Its basic principle is to randomly select n observations with replacement from the original dataset of n observations to generate a new Bootstrap dataset. Then, NONMEM is used to fit the parameters of the Bootstrap dataset, and this process is repeated multiple times for summary analysis. In this study, 1000 resamplings were performed, and the median and 95% confidence interval of the parameters estimated based on the Bootstrap datasets were calculated and compared with the parameter estimates of the final model. The model was considered stable and the parameter estimates accurate if the following criteria

were met: (1) The parameter estimates of the final model fell within the 2.5%-97.5% interval of the Bootstrap parameters; (2) A high proportion of the 1000 Bootstrap datasets successfully estimated the parameters.

3.4.8 Final Model

The final model shall meet the following criteria:

(1) The number of significant digits of all estimated typical parameter (θ) values is ≥ 3;

(2) The relative standard error (RSE) of θ is < 35%;

(3) No unexplainable trends are shown in the Goodness of Fit (GOF) plots.

4. Model Application

Using the established population pharmacokinetic (PopPK) model, steady-state concentration-time profiles of the reference cetuximab formulation (Erbitux®) were simulated for all subjects in the study under different dosing regimens. Steady-state data over a 6-week dosing period were analyzed, corresponding to three administrations for the every-two-weeks (Q2W) regimen and two administrations for the every-three-weeks (Q3W) regimen.

Pharmacokinetic parameters, including $AUC_{0\text{-}\tau,\ ss}$, $C_{max,\ ss}$, and $C_{min,\ ss}$, were derived from the simulated steady-state profiles. $AUC_{0\text{-}\tau,\ ss}$ was defined as the area under the concentration-time curve during one steady-state dosing interval, while $C_{max,\ ss}$ and $C_{min,\ ss}$ were defined as the mean steady-state peak and trough concentrations, respectively, within the 6-week dosing window.

5. Quality Control

Comprehensive quality control procedures will be applied to all stages of the PK analysis and report preparation to ensure that the analytical process is traceable, reproducible, and reliable.

6. Appendix

Software Information for PK Analysis

Software	Version
NONMEM	7.5.0
PIRANA	2.9.2
PSN	5.0.0
R	4.3.0

Supplementary Appendix: Clinical Trial Protocol

Efficacy and Safety of Every Three Weeks Cetuximab in Combination With Capecitabine as First-line Maintenance Treatment for RAS/BRAF Wild-type Metastatic Colorectal Cancer: A Phase Ib Dose-Escalation Study

Clinical Trial Protocol

**Trial No.:**	GIHSYSU-29
**Principal trial unit:**	The Sixth Affiliated Hospital, Sun Yat-sen University
**Principal investigator:**	Yanhong Deng, MD, PhD
**Version:**	Version 1.0

This trial will be conducted in accordance with the International Conference On Harmonization - Good Clinical Practice (ICH-GCP) guidelines.

Protocol synopsis

**Trial title**	Efficacy and safety of every three weeks (Q3W) cetuximab in combination with capecitabine as first-line maintenance treatment for RAS/BRAF wild-type metastatic colorectal cancer: a phase Ib dose-escalation study
**Trial No.**	GIHSYSU-29
**Principal investigator**	Yanhong Deng, MD, PhD<br>The Sixth Affiliated Hospital, Sun Yat-sen University
**Trial design**	This study was an investigator-initiated, single-center, dose-escalation phase Ib trial.
**Target populations**	Patients with metastatic colorectal cancer who are RAS and BRAF wild-type. Subjects who have completed at least eight cycles of first-line induction therapy (cetuximab + FOLFOX/ FOLFIRI, Q2W) and have not experienced disease progression (CR/PR or SD) will be screened.
**Inclusion criteria**	1. Men and women aged ≥18 years old with an expected life span of at least 12 weeks at the time of signing the Informed Consent Form (ICF).<br>2. Histologically or cytologically confirmed RAS and BRAF wild-type metastatic colorectal adenocarcinoma (mCRC), excluding appendiceal and anal cancers.<br>3. Completed at least eight cycles of first-line induction therapy (cetuximab + FOLFOX/ FOLFIRI, Q2W) and have not experienced disease progression (CR/PR or SD).<br>4. Eastern Cooperative Oncology Group (ECOG) performance status of 0-1.<br>5. Adequate haematological, hepatic, and renal function: Hemoglobin ≥ 90g/L, neutrophil count ≥ 1.5 × 10^9/L, platelet count ≥ 75 × 10^9/L; Serum total bilirubin ≤ 1.5 × upper limit of normal (UNL); Aspartate aminotransferase (AST) or alanine aminotransferase (ALT) ≤ 2.5 × UNL; if there are liver metastases, AST or ALT ≤ 5 × UNL; Serum creatinine ≤ 1.5 × UNL.
**Exclusion criteria**	1. Disease progression occurred after adjuvant therapy within 6 months (for chemotherapy not containing oxaliplatin) or within 9 months (for chemotherapy containing oxaliplatin).<br>2. Concurrent active malignancy, excluding malignancies with disease-free survival of 5 years or more or in situ carcinoma considered cured after adequate treatment.<br>3. Known brain metastases or leptomeningeal metastases. Patients with neurological symptoms should undergo brain CT/MRI to exclude metastases.<br>4. Patients with any Grade 2 or above toxicity as classified by the common terminology criteria for adverse events (CTCAE) (version 5.0) (except for anemia, alopecia and skin pigmentation) which is induced by previous treatment and has not subside. Patients with CTCAE Grade 3 or above neurotoxicity caused by platinum-based drugs should be excluded.<br>5. Ascites, pleural effusion, or pericardial effusion requiring drainage within the past 4 weeks.<br>6. Bowel obstruction, gastrointestinal bleeding, pulmonary fibrosis or

	interstitial pneumonia, renal failure, liver failure, or cerebrovascular disease.<br>7. Uncontrolled diabetes or uncontrolled hypertension.<br>8. Myocardial infarction within the past 12 months, severe/unstable angina pectoris, or New York Heart Association (NYHA) Class III or IV congestive heart failure symptoms.<br>9. History of known or suspected allergies to any related drugs used in the trial.<br>10. Known infection with human immunodeficiency virus (HIV), acquired immunodeficiency syndrome (AIDS) related diseases, hepatitis B or C.<br>11. Autoimmune diseases or a history of organ transplantation requiring immunosuppressive therapy.<br>12. Mental illness that may increase the risk associated with participation in the study or interfere with the interpretation of study results.<br>13. Major surgery or radiotherapy within 4 weeks.<br>14. Women who are pregnant or nursing.<br>15. Presence of any other serious illness.
**Study endpoints**	**Primary endpoint:**<br>● Pharmacokinetic (PK) characteristics<br>● Safety<br>● Maximum tolerated dose (MTD)<br>**Secondary endpoints:**<br>● Progression-free survival (PFS)<br>● Overall Survival (OS)<br>● Objective response rate (ORR)<br>● Disease Control Rate (DCR)<br>● Quality of Life (QOL)<br>**Other Pre-specified Outcomes**<br>● Treatment-emergent genetic mutations through ctDNA testing
**Investigational drugs**	Cetuximab and capecitabine
**Treatment regimen**	Eligible patients received cetuximab at escalating doses of 400 mg/m², 500 mg/m², 600 mg/m², and 700 mg/m² every three weeks (Q3W), in combination with fixed-dose oral capecitabine administered at 1000 mg/m² twice daily on days 1-14 of each 21-day cycle. Maintenance therapy was continued every three weeks (Q3W) until disease progression, the occurrence of dose-limiting toxicities (DLTs), or determination of the maximum tolerated dose (MTD). Patients who experienced unacceptable toxicity related to chemotherapy were permitted to continue cetuximab monotherapy at the investigator's discretion.
**Sample size**	This is a phase Ib dose-escalation study that adopted a traditional 3+3 dose-escalation design. If none of the patients treated at the starting dose level experienced a dose-limiting toxicity (DLT), successive cohorts of three fully evaluable patients were enrolled at the next cetuximab dose levels. If one of three patients experiences a dose-limiting toxicity (DLT), expand the

	cohort to six patients: If ≤1 of six patients have dose-limiting toxicities (DLTs), escalate.If ≥2 of six have dose-limiting toxicities (DLTs), halt escalation and declare the previous dose as maximum tolerated dose (MTD). If ≥2 of the initial three patients have DLTs, the MTD is exceeded, and the prior dose level is expanded to confirm MTD. The estimated sample size is 3 to 24.
**Trial duration**	From February, 2023 to July, 2025

1. Background

Colorectal cancer (CRC) is the third most common cancer and second leading cause of cancer-related death worldwide.[1] Targeted therapies have transformed the treatment landscape for cancer patients in recent years by offering greater precision than traditional cytotoxic chemotherapies. Fluorouracil-based chemotherapy (in combination with oxaliplatin or irinotecan) plus anti-epidermal growth factor receptor/ vascular endothelial growth factor (anti-EGFR/VEGF) therapy is the standard first-line treatment regimen for metastatic colorectal cancer (mCRC).[2-6] After achieving disease stabilization or a more favorable response, switching to low-intensity or low-toxicity maintenance therapy can balance clinical efficacy and adverse events (AEs). OPTIMOX-1 found that a maintenance regimen was as effective as continuous therapy while significantly reducing adverse events (AEs) and improving quality of life (QoL).[7] Building on this, OPTIMOX-2 confirmed that maintenance therapy was superior to an intermittent approach in patients with unresectable disease.[8] The results of these trials suggest that a single cytotoxic agent may be a valuable maintenance option following intensive induction therapy. With the advent of the era of precision medicine, targeted therapy has become one of the key treatment modalities for metastatic colorectal cancer (mCRC). The Stop and Go and the CAIRO3 study suggest that bevacizumab combined with capecitabine maintenance therapy offers a progression-free survival (PFS) advantage.[9-10] For instance, in the CAIRO3 study, the median progression-free survival (PFS) in the maintenance group was 11.7 months, compared with 8.5 months in the observation group (HR = 0.67, $P < 0.0001$). In terms of QoL, there was no statistically significant difference between the maintenance and observation group. Currently, this combination maintenance therapy regimen is widely adopted in clinical practice.

For patients with RAS/BRAF wild-type mCRC, particularly those with left-sided tumors, studies like FIRE-3 and CALGB/SWOG 80405 have demonstrated that continuous treatment with cetuximab plus chemotherapy until disease progression is both effective and feasible.[3, 6] This regimen yields superior outcomes compared to bevacizumab combined with chemotherapy. Given cetuximab's high efficacy and low toxicity, its role in maintenance therapy is also being actively and continuously explored. Evidence from several key clinical trials, including COIN-B, MACRO-2, NORDIC VII and PRODIGE 28, collectively supports the use of cetuximab as a maintenance therapy.[11-14] Moreover, the MACBETH trial evaluated maintenance therapy with cetuximab or bevacizumab following induction therapy that included chemotherapy combined with cetuximab.[15] The results indicated that the cetuximab group achieved superior clinical efficacy; however, this difference was not statistically significant. The VALENTINO and PANAMA trial investigated a maintenance strategy using anti-EGFR monoclonal antibodies combined with single-agent chemotherapy.[16-17] The results demonstrated that adding the EGFR-targeted drug panitumumab to 5-fluorouracil(5-FU) as a maintenance regimen significantly improved PFS and overall survival (OS) compared to panitumumab monotherapy. In addition, the TJCC005 study confirmed the high efficacy of maintenance therapy combining capecitabine with cetuximab, demonstrating a median PFS of 12.7 months and a median overall survival (OS) of 27.4 months.[18] Another study showed that maintenance therapy with cetuximab combined with irinotecan significantly prolonged failure-free survival compared to discontinuing treatment.[19]

Based on current research, a common strategy for maintenance therapy in RAS/BRAF wild-type mCRC is to combine cetuximab with a single cytotoxic drug like 5-FU. However, this regimen presents logistical challenges that can compromise its long-term suitability. Because 5-FU is an intravenous drug and cetuximab are administered weekly or biweekly, patients require frequent hospital visits. This increases treatment costs and reduces convenience, making the approach less than ideal for an ongoing

maintenance therapy model. An optimized, every-3-week dosing schedule for cetuximab, combined with oral capecitabine, may provide an alternative maintenance therapy for patients with RAS/BRAF wild-type mCRC who have completed induction chemotherapy with cetuximab. Therefore, we conducted a prospective phase Ib clinical trial to evaluate the pharmacokinetic (PK) characteristics, assess the safety, and determine the MTD of this regimen.

2. Potential risks and benefits

2.1 Potential risks

Any drug or therapy used in any stage of the trial may result in unforeseen or even serious side effects. For patients with metastatic colorectal cancer receiving capecitabine (XELODA®) monotherapy, a remarkably high 96% experienced at least one adverse event. The most frequently reported adverse reactions (≥10% incidence) included gastrointestinal issues such as diarrhea (55%), nausea (43%), abdominal pain (35%), vomiting (27%), and stomatitis (25%), alongside Hand-and-Foot Syndrome (54%), fatigue/weakness (42%), and hyperbilirubinemia (48%). Notably, anemia was reported in 80% of patients, and neutropenia in 13%. Other common reactions included decreased appetite (26%), dermatitis (27%), pyrexia (18%), edema (15%), eye irritation (13%), and dyspnea (14%). While gastrointestinal and dermatologic toxicities remain prominent, the data for metastatic colorectal cancer reveals an exceptionally high incidence of Anemia (80%) and Hyperbilirubinemia (48%). The hyperbilirubinemia is particularly concerning given its Grade 3 (18%) and Grade 4 (5%) rates. Grade 3 or 4 hyperbilirubinemia occurred in 22.8% of patients with hepatic metastases at baseline, compared to 12.3% without. This indicates that in patients with metastatic colorectal cancer, often characterized by liver involvement, hepatic and hematologic monitoring must be a high priority. The elevated risk of severe hyperbilirubinemia, especially in the presence of liver metastases, necessitates regular liver function tests and prompt dose interruption if significant elevations occur. Anemia management, while often less immediately critical than acute toxicities, is vital for maintaining patient quality of life and performance status throughout prolonged treatment.

According to the cetuximab (ERBITUX®) instruction manual, for patients with advanced colorectal cancer, adverse reactions are highly prevalent across various treatment regimens. The spectrum of adverse reactions with an incidence of 10% or greater consistently includes prominent dermatologic toxicities such as acneiform rash (reported as high as 95% for single-agent use and 88% in combination with irinotecan), dry skin (up to 57%), and pruritus (up to 47%). Gastrointestinal disturbances are also very common, with diarrhea (up to 72% in combination with irinotecan, 66% with FOLFIRI, 55% single-agent), nausea (up to 64% single-agent, 55% with irinotecan), vomiting (up to 40% single-agent), abdominal pain (up to 35% single-agent), and stomatitis (up to 32% single-agent) frequently observed. Other prevalent adverse reactions include fatigue/asthenia (up to 91% single-agent, 73% with irinotecan, 51% with encorafenib), headache (up to 38% single-agent), decreased appetite (up to 27%), and various neurological symptoms like peripheral sensory neuropathy (up to 45% single-agent). The specific incidence and severity of these reactions can vary depending on the combination therapy used. Severe adverse reactions (Grade 3 or 4) in advanced colorectal cancer patients commonly include dermatologic toxicities such as acne-like rash (up to 18% with FOLFIRI) and rash/desquamation (16% with single-agent), along with significant gastrointestinal issues like diarrhea (up to 22% with irinotecan, 16% with FOLFIRI) and nausea/vomiting (up to 6% and 5% respectively with single-agent). Hematologic toxicities, particularly neutropenia (31% with FOLFIRI) and leukopenia (17% with irinotecan), are also notable. General symptoms like fatigue/asthenia (up to 31% with single-agent, 16% with irinotecan) and pain (up to 18% with single-agent) are frequently severe. Other significant

Grade 3/4 events include infections, dehydration, and infusion reactions, with specific incidences varying by the combination regimen used.

The combined use of cetuximab (ERBITUX®) and capecitabine (XELODA®), typically as components of multi-drug regimens in cancer treatment, presents a complex yet generally manageable toxicity profile. Most of the adverse reactions are mild to moderate (Grade 1-2). The most prominent and frequently high-grade adverse reactions observed are gastrointestinal (e.g., diarrhea, stomatitis) and dermatologic (e.g., acneiform rash, hand-foot syndrome). These are largely attributable to the overlapping mechanisms of action of both agents, which can lead to an amplified incidence and severity of these side effects. Infusion reactions, specific to cetuximab (ERBITUX®), remain a critical concern, with evidence suggesting a potentially higher incidence of severe reactions in combination settings, which requires particular attention. Furthermore, hematologic toxicities and electrolyte imbalances are important considerations that necessitate careful monitoring. They can be recovered by symptomatic treatment or suspension of medication, and have relatively small effect on the patient's physiological function and quality of life without hindering the continuation of the trial. Symptomatic treatment of the adverse reactions according to supportive care NCCN Clinical Practice Guidelines can ensure the safety of patients during the trial.

2.2 Potential benefits

Current clinical trial data suggests that the combined use of cetuximab and capecitabine offers a promising maintenance therapy option for mCRC. This combination has demonstrated the ability to significantly improve progression-free survival and overall prognosis in patients with wild-type RAS and BRAF tumors. The observed toxicities, which can include rash and hand-foot syndrome, are generally mild and manageable, allowing for a favorable safety profile that supports long-term use. A three-week dosing regimen for this combination further enhances its potential benefits. This schedule simplifies the treatment plan by reducing the frequency of hospital visits for cetuximab infusions, which in turn lowers the overall burden and cost of care for patients. This improved convenience and potential for reduced toxicities, without compromising the clinical efficacy, contribute to a better quality of life during treatment.

3. Trial design and objective

3.1 Overall trial design

This study was an investigator-initiated, single-center, dose-escalation phase Ib trial. We employed a standard 3+3 dose-escalation design. It is intended to enroll 3 to 24 patients with RAS and BRAF wild-type mCRC. Subjects who have completed at least eight cycles of first-line induction therapy (cetuximab + FOLFOX/ FOLFIRI, Q2W) and have not experienced disease progression (CR/PR or SD) will be screened. Eligible patients received cetuximab at escalating doses of 400 mg/m², 500 mg/m², 600 mg/m², and 700 mg/m² every three weeks (Q3W), in combination with fixed-dose oral capecitabine administered at 1000 mg/m² twice daily on days 1-14 of each 21-day cycle. Maintenance therapy was continued Q3W until disease progression, the occurrence of DLTs, or determination of the MTD. Patients who experienced unacceptable toxicity related to chemotherapy were permitted to continue cetuximab monotherapy at the investigator's discretion.

If none of the patients treated at the starting dose level experienced a DLT, successive cohorts of three fully evaluable patients were enrolled at the next cetuximab dose levels. If one of three patients experiences a DLT, expand the cohort to six patients: If ≤1 of six patients have DLTs, escalate.If ≥2 of six have DLTs, halt escalation and declare the previous dose as MTD. If ≥2 of the initial three patients have DLTs, the MTD is exceeded, and the prior dose level is expanded to confirm MTD. Intrapatient

dose escalation was not permitted. The MTD is the highest dose where ≤1/6 patients experience DLTs. DLT was defined as any grade 3 or 4 haematological or non-haematological toxicity or administration of <66% of the assigned dose (i.e. a delay of >14 days in the first 6 weeks) due to toxicity at any of the cetuximab doses.

To further evaluate the pharmacokinetic (PK) and efficacy comparability between the dose-escalation regimens and the standard dosing regimen of cetuximab at 500 mg/m^2 every-two-weeks (Q2W), an additional six patients were enrolled.

For the pharmacokinetic (PK) analysis of cetuximab, 4.0 mL blood samples were collected from patients before and at the end of cetuximab infusion during cycles 1 to 4. Serum was extracted from blood immediately and stored at -80°C until analysis.

3.2 Study flow diagram

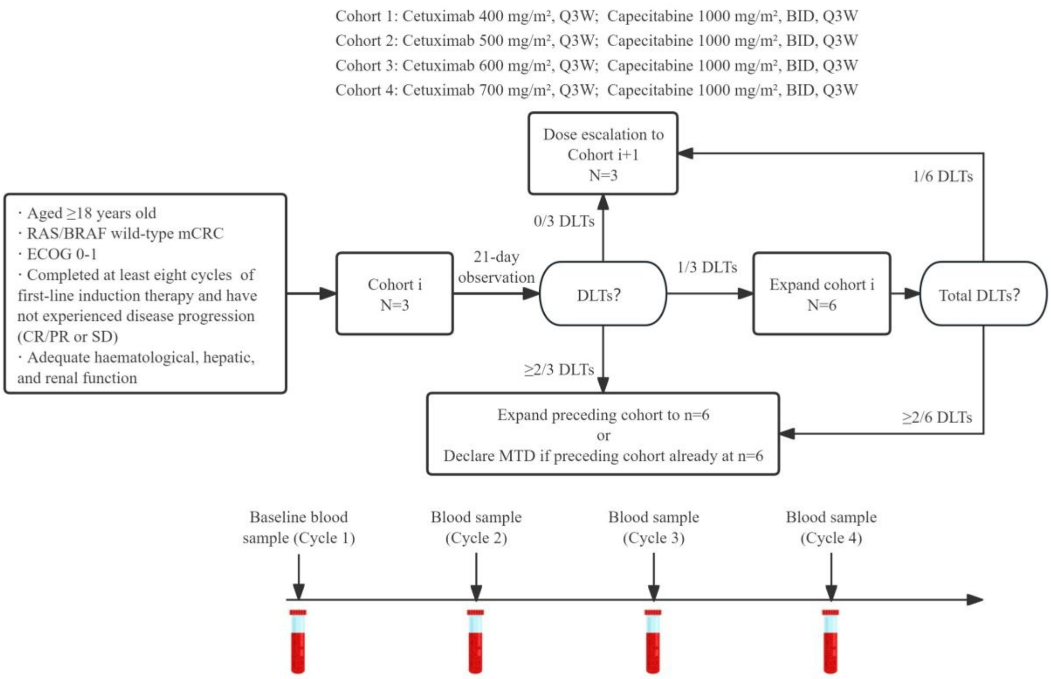

3.3 Trial objectives

Primary objectives

To evaluate the PK characteristics, assess safety, and determine the MTD of cetuximab in combination with capecitabine every three week on the basis of the occurrence of DLT.

Secondary objectives

To evaluate the efficacy parameters including PFS, OS, ORR, DCR and QOL of cetuximab in combination with capecitabine as first-line maintenance therapy in patients with RAS/BRAF wild-type mCRC.

Exploratory objective

Monitoring changes in genetic mutations during cetuximab treatment using circulating tumor DNA (ctDNA) analysis.

3.4 Study endpoints

3.4.1 Primary endpoints

- PK characteristics
- Safety

- MTD

3.4.2 Secondary endpoints

- PFS
- OS
- ORR
- DCR
- QOL

3.4.3 Exploratory endpoints

- Treatment-emergent genetic mutations through ctDNA testing

4. Selection and withdrawal of subjects

4.1 Inclusion criteria

Patients who meet all of the following inclusion criteria are eligible to participate in this trial:

- Provide a written informed consent form (ICF) before any research procedure is carried out.
- Patients must be ≥18 years old and have an expected life span of at least 12 weeks when signing the ICF.
- Patients have histologically or cytologically confirmed RAS and BRAF wild-type mCRC, excluding appendiceal and anal cancers.
- After being diagnosed with mCRC, patients have only received cetuximab combined with chemotherapy (FOLFOX or FOLFIRI) as first-line induction therapy. Imaging progression during adjuvant therapy or within 6 months after completion of adjuvant therapy is considered as first-line treatment.
- Patients have completed at least eight cycles of cetuximab combined with chemotherapy induction therapy and the disease is controlled (including CR/PR and SD).
- There is at least one measurable metastatic lesion, defined as per RECIST version 1.1. Patients who have achieved CR without measurable lesions after induction therapy, and those who have achieved no evidence of disease (NED) through R0 resection, interventional ablation, or other local destructive therapies can be included in this study.
- Eastern Cooperative Oncology Group (ECOG) performance status of 0-1.
- Within 7 days before treatment, the following laboratory test values are obtained and appropriate organ function is present: Hemoglobin ≥ 90g/L, neutrophil count ≥ 1.5 × 10^9/L, platelet count ≥ 75 × 10^9/L; Serum total bilirubin ≤ 1.5 × upper limit of normal (UNL); Aspartate aminotransferase (AST) or alanine aminotransferase (ALT) ≤ 2.5 × UNL; if there are liver metastases, AST or ALT ≤ 5 × UNL; Serum creatinine ≤ 1.5 × UNL.
- Patients are not allowed to participate in other clinical trials during the study period.
- Patients are willing and able to comply with the study protocol and visit plan.

4.2 Exclusion criteria

- Disease progression occurred after adjuvant therapy within 6 months (for chemotherapy not containing oxaliplatin) or within 9 months (for chemotherapy containing oxaliplatin).
- Concurrent active malignancy, excluding malignancies with disease-free survival of 5 years or more or in situ carcinoma considered cured after adequate treatment.
- Known brain metastases or leptomeningeal metastases. Patients with neurological symptoms should undergo brain CT/MRI to exclude metastases.
- Patients with any Grade 2 or above toxicity as classified by the common terminology criteria for adverse events (CTCAE) (version 5.0) (except for anemia, alopecia and skin pigmentation) which

is induced by previous treatment and has not subside. Patients with CTCAE Grade 3 or above neurotoxicity caused by platinum-based drugs should be excluded.

- Ascites, pleural effusion, or pericardial effusion requiring drainage within the past 4 weeks.
- Patients with bowel obstruction, gastrointestinal bleeding, pulmonary fibrosis or interstitial pneumonia, renal failure, liver failure, or cerebrovascular disease.
- Uncontrolled diabetes, defined as HbA1c >7.5% after the use of antidiabetic drugs, or uncontrolled hypertension, defined as systolic/diastolic blood pressure > 140/90mmHg after the use of antihypertensive drugs.
- Myocardial infarction within the past 12 months, severe/unstable angina pectoris, or New York Heart Association (NYHA) Class III or IV congestive heart failure symptoms.
- A history of allergy to any study drugs (such as cetuximab or capecitabine).
- Known infection with human immunodeficiency virus (HIV), acquired immunodeficiency syndrome (AIDS) related diseases, hepatitis B or C.
- Autoimmune diseases or a history of organ transplantation requiring immunosuppressive therapy.
- Mental illness that may increase the risk associated with participation in the study or interfere with the interpretation of study results.
- Received any of the following treatments within a specified time period prior to receiving the study drug: Major surgery within 4 weeks (excluding diagnostic biopsy, surgical incision should be completely healed before administering the study drug); Radiotherapy within 4 weeks; Other anti-tumor treatments or participation in other clinical trials within 4 weeks, except for induction therapy as specified in the protocol.
- Pregnant (confirmed by serum human chorionic gonadotropin [hCG] test) or lactating women, or women of childbearing potential who plan to become pregnant during the treatment period and within 2 months after the end of cetuximab treatment, or within 6 months after the end of capecitabine treatment. Women of childbearing potential or sexually active men who are unwilling to use contraception during the study period and for at least 2 months after the end of cetuximab treatment, or 6 months after the end of capecitabine treatment. Postmenopausal women must have been amenorrheic for at least 12 months to be considered of non-childbearing potential.
- Presence of any other serious illness that, in the investigator's opinion, would preclude the patient's participation in the study.

4.3 Criteria for withdrawing from the trial

Subjects can voluntarily withdraw from the trial at any time, or be requested by the investigator to withdraw from the trial due to safety or behavior concerns or failure in following the visits or procedures conducted in the trial center where they are involved in the study as required in the protocol.

5. Use of investigational drugs

Usage, dosage adjustment, delayed administration and termination of the administration of cetuximab and capecitabine

Eligible patients received cetuximab at escalating doses of 400 mg/m², 500 mg/m², 600 mg/m², and 700 mg/m² Q3W. Capecitabine was administered at a fixed dose of 1000 mg/m² twice daily from days 1 to 14 of each 21-day treatment cycle. Dose modifications were not permitted, but cetuximab and/or capecitabine could be interrupted or delayed for a maximum of 2 weeks. Subjects who experience a DLT will be immediately withdrawn from treatment and entered into the follow-up period.

An additional six patients received the standard regimen, which included cetuximab (500 mg/m²,

2-hour infusion), folinic acid (400 mg/m² racemic or 200 mg/m² L-form, 2-hour infusion), followed by 5-fluorouracil (5-FU) administered as a 400 mg/m² intravenous bolus and a 46-hour continuous infusion of 2400 mg/m².

6. Combined medication and concomitant medication

6.1 Allowed combined medication and concomitant medication

Supportive care or other necessary treatments were permitted to ensure patient safety. All concomitant medications administered throughout the study were documented. In the event of an adverse reaction, appropriate medical management was provided, and all medications used for its treatment were recorded and specified.

External application, spray inhalation, eye drops or topical application of corticosteroids is allowed. Short-term (not more than 3 weeks) use of corticosteroids to treat non-autoimmune diseases (e.g., delayed allergic reactions caused by contact allergens) is allowed.

6.2 Prohibited combined medication and concomitant medication

- No anti-tumor agents other than the investigational drug may be administered, including, but not limited to, chemotherapy, immunotherapy, targeted therapy, biological therapy, hormone therapy, non-palliative radiotherapy and traditional Chinese medicines with anti-tumor indications.
- Live vaccines are prohibited during the study. Inactivated or attenuated influenza vaccines may be administered. The use of other inactivated or attenuated vaccines for infectious disease prevention must be determined based on clinical circumstances and discussed with the investigators.
- It is prohibited to use strong inducers or inhibitors of the drug-metabolizing enzyme CYP3A4.
- The use of herbal medicines is prohibited.

6.3 Other anti-tumor treatments and experimental drugs

During the trial period, subjects are not allowed to receive other anti-tumor treatments not specifically specified in this trial protocol. They are also not allowed to receive other systemic anti-tumor treatments.

7. Common treatment-related adverse reactions and symptomatic treatment recommendations

7.1 Common cetuximab-related adverse reactions and symptomatic treatment recommendations

Common cetuximab-related adverse reactions (incidence ≥25%) include various cutaneous reactions such as rash, pruritus, and nail changes, along with headache, diarrhea, and infection. When cetuximab is combined with other chemotherapy regimens such as FOLFIRI, irinotecan, 5-fluorouracil/platinum, or encorafenib, the most common adverse reactions (incidence ≥25%) include cutaneous adverse reactions (rash, pruritus, nail changes, dermatitis acneiform), headache, diarrhea, infection, fatigue, nausea, abdominal pain, decreased appetite, and arthralgia.

- Dermatologic Toxicities (e.g., Acneiform Rash): Patients should limit sun exposure during treatment and for two months after the last dose. For Grade 1-2 reactions, symptomatic treatments such as topical or oral antibiotics can be used while continuing cetuximab. For Grade 3 or 4 reactions, cetuximab must be immediately discontinued and appropriate supportive therapies administered.
- Electrolyte Abnormalities (e.g., Hypomagnesemia, Hypokalemia, Hypocalcemia): These can occur days to months after starting cetuximab. Serum electrolytes should be monitored weekly during treatment and for at least 8 weeks after completion, with repletion as necessary.
- Pulmonary Toxicity (e.g., Interstitial Lung Disease): Patients should be monitored for new or worsening pulmonary symptoms. If acute onset or worsening symptoms occur, the infusion

should be delayed. If there's no improvement within two weeks or if interstitial lung disease is confirmed, cetuximab should be permanently discontinued.

- Infusion Reactions: To prevent these, patients should be premedicated with a histamine-1 (H1) receptor antagonist intravenously 30-60 minutes before the first and subsequent doses. For Grade 1 or 2 reactions, the infusion rate should be reduced by 50%. For Grade 2 reactions, the infusion should be stopped, supportive therapies administered, and then resumed at 50% of the previous rate once resolved to Grade 1 or below. For Grade 3 or 4 reactions, cetuximab must be immediately and permanently discontinued, and appropriate supportive therapies administered.
- Cardiopulmonary Arrest: Serum electrolytes, including magnesium, potassium, and calcium, should be closely monitored during and after cetuximab administration.
- Fatigue/Asthenia: These general symptoms are frequently observed. Management typically involves supportive care and may require dose adjustments of the chemotherapy agents if severe.
- Nausea/Vomiting: These gastrointestinal symptoms are common. Symptomatic treatment with antiemetics is recommended.

7.2 Common capecitabine-related adverse reactions and symptomatic treatment recommendations

The most common adverse reactions (occurring in 30% or more of patients) associated with capecitabine are diarrhea, hand-and-foot syndrome, nausea, vomiting, abdominal pain, fatigue/weakness, and hyperbilirubinemia.

- Diarrhea (Grade 3 or above): Capecitabine should be immediately interrupted until it resolves or decreases to Grade 1, and standard antidiarrheal treatments like loperamide are recommended. Patients with severe diarrhea should receive fluid and electrolyte replacement.
- Hand-and-foot syndrome (Grade 3 or above): Capecitabine treatment should be interrupted until symptoms resolve or decrease to Grade 1. Symptomatic treatment is also recommended.
- Nausea or vomiting (Grade 3 or above): Capecitabine should be immediately interrupted, and symptomatic antiemetic treatment initiated.
- Hyperbilirubinemia (Grade 3 or above): Capecitabine should be interrupted until bilirubin levels decrease to $\leq 3.0 \times$ UNL.
- Dehydration occurs (Grade 3 or above): Capecitabine should be interrupted, and dehydration corrected, with treatment resuming only after rehydration and control of precipitating causes.
- Stomatitis (Grade 3 or above): Capecitabine should be interrupted, and symptomatic treatment initiated.
- Fever and neutropenia: Patients should contact their physician immediately, and if Grade 3 or 4 neutropenia or thrombocytopenia occurs, therapy should be stopped until the condition resolves.

8. Trial process

8.1 Screening period

The screening period starts with the signing of the informed consent form and ends with the first time of receiving the investigational drug or failure of screening.

Patients must sign an informed consent form before proceeding to the screening procedures specified in this trial. If laboratory examinations and imaging evaluations required for routine clinical diagnosis and treatment are performed before the informed consent form is signed, relevant data can be used if they are within the specified window period.

The following screening should be completed within 14 days before the start of the treatment with the investigational drug:

- Obtain the signed informed consent form from the subject.
- Collect demographic data, including the name, gender, date of birth, height, weight, etc.
- Imaging examination: CT of the chest, the whole abdomen and the pelvic cavity.
- Tumor diagnosis: date of pathological diagnosis, pathological grade, clinical imaging stage (TNM), etc.
- RAS/BRAF gene detection of tumor tissues: Prior to the initiation of first-line induction therapy, RAS/BRAF gene status was determined from biopsy or surgical pathological specimens of primary or metastatic lesions. The analysis was performed using either a PCR-based assay or next-generation sequencing (NGS). Genetic testing was performed to identify mutations in the following loci: KRAS exons 2 (codons 12 and 13), 3 (codons 59 and 61), and 4 (codons 117 and 146); NRAS exon 2 (codons 12 and 13), exon 3 (codons 59 and 61), and exon 4 (codons 117 and 146); BRAF exon 15 (codon 600). Patients with a previously undetermined RAS/BRAF gene status were required to undergo testing before the start of maintenance therapy. If the gene status had already been established prior to first-line induction therapy, retesting was not necessary.

The following screening should be completed within 7 days before the start of the treatment with the investigational drug:

- Weight and ECOG score.
- Vital signs: pulse, respiratory rate, body temperature and blood pressure.
- Comprehensive physical examination: general condition, head and face, skin, lymph nodes, eyes, ears, nose, throat, oral cavity, respiratory system, cardiovascular system, abdomen, reproductive-urinary system, musculoskeletal system, nervous system, mental state, etc.
- Routine blood tests: red blood cell count, hemoglobin, platelet count, white blood cell count, neutrophil count and differential lymphocyte count.
- Routine urine tests: White blood cells, red blood cells, urine protein; if urine protein ≥ 2+, quantitative detection of 24-hour urine protein must also be conducted.
- Blood biochemistry: alanine aminotransferase (ALT), aspartate aminotransferase

(AST), γ-glutamyl transpeptidase (γ-GT), total bilirubin (TBIL), direct bilirubin (DBIL), alkaline phosphatase (AKP), blood urea nitrogen (BUN) or urea (preferably blood urea nitrogen), total protein (TP), albumin (ALB), creatinine (Cr), blood glucose (GLU), K^+, Na^+, Ca^{2+}, Mg^{2+} and Cl^-.

- 12-lead electrocardiogram: QT, QTc and P-R intervals should be noted. If there is any abnormality, other related examinations should be performed based on the evaluation of the investigator.
- Echocardiography: At least an assessment of left ventricular ejection fraction (LVEF) should be included.
- Pregnancy test: Suitable for women of childbearing age, using serum pregnancy test.

8.2 Randomization and masking

This Phase Ib trial utilized a dose-escalation design and did not involve patient randomization. Investigators who assessed the radiological response were masked to the treatment group assignment and all other data.

8.3 Treatment period

- Based on the previously described study design, subjects were enrolled into different cetuximab dose-escalation groups for treatment. The first cohort of three fully evaluable patients in the dose-escalation phase received cetuximab at an initial dose of 400 mg/m² Q3W, combined with fixed-dose capecitabine at 1000 mg/m² twice daily on days 1-14 of each cycle. If none of the patients treated at the starting dose level experienced a DLT, successive cohorts of three fully

evaluable patients were enrolled at the next cetuximab dose levels: 500mg/m², 600mg/m², and 700 mg/m². If one of three patients experiences a DLT, expand the cohort to six patients: If ≤1 of six patients have DLTs, escalate. If ≥2 of six have DLTs, halt escalation and declare the previous dose as MTD. If ≥2 of the initial three patients have DLTs, the MTD is exceeded, and the prior dose level is expanded to confirm MTD.

- Monitoring of vital signs and a comprehensive physical examination should be performed on the day before administration of cetuximab and capecitabine.
- Routine blood test, blood biochemistry, routine urine test and electrocardiography should be completed within 3 days before administration of cetuximab and capecitabine in each cycle.
- CT of the chest, the whole abdomen and the pelvic cavity should be performed within 14 days following every third cycle of cetuximab and capecitabine administration.

8.4 Termination of the study treatment

Termination of the study treatment does not mean withdrawal from the study. Subjects who terminate the study treatment should continue to complete the remaining study visits as required by the protocol. If any of the following occurs, the subject must terminate administration of the investigational drug:

- The subject requests the termination of the treatment with the investigational drug.
- Medical imaging or clinical symptoms suggest disease progression.
- The subject becomes pregnant during the study.
- There is any clinical adverse event (AE), laboratory abnormalities or other medical conditions due to which the subject may no longer benefit from continuing the study.
- The health condition of the subject is generally deteriorated, which makes the subject fail to continue participating in the trial.
- Significant deviations from the protocol, such as non-conformity and non-compliance, are found after the subject is enrolled in the group.

8.5 Toxicity assessment

The investigator will record the number (%) of patients with AEs and the type of AEs from the first medication of the investigational drug to 90 days after the last medication and classify the AEs according to CTCAE v.5.0.

8.6 Tumor assessment

Radiological tumor assessment

Thoracoabdominopelvic CT scans and/or pelvic MR will be used to evaluate the tumor response. The assessment in the screening period/baseline assessment should be carried out within 14 days before the date of the first administration of cetuximab and capecitabine. Following the screening assessment, subsequent efficacy evaluations were to be conducted within 14 days after the completion of every third cycle of cetuximab and capecitabine administration. Change of tumor volume and the maximum diameter were evaluated by a trained radiologist, who was masked to the treatment assignment and all other data.

8.7 Collection and storage of Blood specimen

For pharmacokinetic (PK) analysis of cetuximab, 4.0 mL blood samples were collected from patients within 1 hour prior to and immediately after cetuximab infusion during treatment cycles 1 through 4. Whole blood was collected into a Becton Dickinson serum-separating tube (SST), promptly processed to isolate serum, and stored at −80 °C until analysis.

With informed consent, circulating tumor DNA (ctDNA) analysis was performed on 8.0 mL blood samples collected into Cell-Free DNA BCT® tubes (Streck) and processed according to the

manufacturer's instructions. ctDNA samples were collected at three time points: baseline, after confirmed response, and at the time of disease progression. The purpose of this analysis was to assess the association between acquired gene mutations and treatment efficacy.

8.8 Safety assessment

The investigator will continuously monitor and assess treatment-related safety from the time of informed consent through 90 days after the final dose of the investigational drug.

8.9 Survival follow-up

Following completion of treatment or withdrawal from the study, patients entered the survival follow-up phase. Follow-up assessments were conducted every three months until death, loss to follow-up, or study termination. Follow-up methods included outpatient visits, telephone interviews, or review of medical records.

9. Safety evaluation

9.1 Definition of adverse events

An adverse event (AE) refers to any adverse medical event that occurs in a patient or a subject of the clinical trial after the drug is taken, but the event is not necessarily causally related to the treatment received. Therefore, AEs can be any undesirable and unexpected signs (including abnormal laboratory findings) and symptoms, or diseases that are time-related to the use of medical products, regardless of whether they are considered to be related to the investigational drug, at least including the following several conditions:

- Exacerbation of the original (before participating in the clinical trial) medical condition/disease (including exacerbation of symptoms, signs or laboratory abnormalities).
- Any newly-occurring adverse medical conditions (including symptoms, signs or newly diagnosed diseases).
- Abnormal laboratory test values or results with clinical significance.

The investigator should record any AE occurring in the subject in detail, including description of AEs and all related symptoms, time of occurrence, severity, correlation with the investigational drug, duration, measures taken and final results and outcomes. Tumor progression or deterioration (including the appearance of new metastases and death due to disease progression) observed in the trial should be served as a part of the efficacy evaluation and should not be reported as an AE or serious adverse event (SAE).

9.2 Definition of serious adverse events

A serious adverse event (SAE) refers to any adverse medical condition that meets one of the following criteria at any dose:

- Fatal (leading to death; note: death is an outcome, not an event)
- Life-threatening (note: ‘Life-threatening’ means that the patient is in immediate danger of death when the event occurs, rather than the assumption that if the event is more serious, it will cause death)
- Lead to hospitalization or prolonged hospital stay
- Lead to lifelong or severe disability/functional impairment
- Lead to congenital malformations or birth defects
- Have medical significance or require interventions to prevent any of the above consequences

9.3 Classification of AEs and serious adverse events (SAEs)

9.3.1 Evaluating criteria for severity of AEs

The severity of an AE will be graded according to the Common Terminology Criteria for Adverse Events (CTCAE) version 5.0 (http://ctep.cancer.gov/reporting/ctc.html) issued by the National Cancer Institute (NCI) and reported in detail according to the requirements specified in CRF. In the event of an AE that is not included in CTCAE v.5.0, the following five-level scale will be used:

**Mild**	No clinical symptoms or mild clinical symptoms; only clinical or laboratory abnormalities; no need of treatment
**Moderate**	Need of minor, topical or non-invasive treatment; restricted age-appropriate activities of daily living with tools
**Severe**	Severe illness or medically serious symptoms but temporarily not life-threatening; leading to hospitalization or prolonged hospital stay; leading to disability; restriction of self-care in daily life
**Life-threatening**	Immediate life danger requiring urgent medical treatment
**Fatal**	AE-related death

9.3.2 Evaluation of causal relationship between AEs and the investigational drug

In this study, the following criteria will be used to evaluate the causal relationship between AEs and the study treatment: 'definitely related', 'possibly related' and 'cannot be evaluated' will be counted as drug-related adverse reactions, and the incidence of adverse events will be counted.

- Definitely related: The time of occurrence of the reaction conforms to the chronological order of the medication. The reaction conforms to the known reaction type of the investigational drug, and it is improved after dose reduction or drug withdrawal and reappears after resuming administration.
- Possibly related: The time of occurrence of the reaction conforms to the chronological order of the medication. The reaction conforms to the known reaction type of the investigational drug, and it may also be resulted from the patient's clinical state or other treatments.
- Possibly unrelated: The time of occurrence of the reaction does not conform to the chronological order of the medication. The reaction does not quite conform to the known reaction type of the investigational drug and it may also be resulted from the patient's clinical state or other treatments.
- Unrelated: The time of occurrence of the reaction does not conform to the chronological order of the medication. The reaction conforms to the known reaction type of a non-investigational drug, and it may also be resulted from the patient's clinical state or other treatments. Moreover, the reaction disappears once the patient's condition is improved or other treatments are discontinued, and it appears while other treatments are given again.
- Cannot be evaluated: There is no clear relationship between the time of occurrence of the reaction and the chronological order of the medication. The reaction is similar to the known reaction type of the investigational drug, and it may also be resulted from other drugs used at the same time.

9.4 Laboratory abnormalities

The laboratory test results will be recorded on the page showing the laboratory test data in the CRF. The study monitor will review the records in the CRF constantly. Any laboratory abnormality that meets the SAE criteria should be reported immediately using the SAE reporting form, and should also be recorded in the CRF as an AE. Laboratory abnormalities do not have to be reported as AEs on the page describing AEs in the CRF, unless they meet any of the following criteria:

- Accompanied with clinical symptoms;

- The study medication needs to be changed (such as dose adjustment and temporary or permanent discontinuation of dosing);
- Concomitant treatments needs to be changed (e.g., increase the corresponding treatment, interrupt the concomitant treatment or administration, no longer continue or make other changes);
- The laboratory abnormality is believed to have important clinical significance in the investigator's opinion (the investigator should determine whether the independently occurring laboratory abnormality can be classified as an AE based on scientific medical evaluation).

9.5 Follow-up and reporting of AEs

9.5.1 Collection and follow-up of AEs/SAEs

AEs will be collected from the treatment with the investigational drug in the subject to 90 days after the last administration of the investigational drug. AEs/SAEs will be followed up until the event disappears, is relieved to the baseline level or ≤Grade 1, reaches a stable state or gets a reasonable explanation (such as loss to follow-up and death). The optimal outcome should be obtained as much as possible.

9.5.2 Reporting of SAEs

During the study period, no matter what kind of treatment the patient receives, any SAE or any serious abnormal laboratory test result must be filled in AE reporting form and CFDA's SAE reporting form by the investigator, and shall be reported to the designated contact person within 24 hours after it is known:

Contact person for reporting SAEs: GCP Office of the Sixth Affiliated Hospital, Sun Yat-sen University

Contact person in the unit of the research group leader: Deng Yanhong (the Sixth Affiliated Hospital of Sun Yat-sen University)

Tel: 020-38254084

Fax: 020-38254084

E-mail: dengyanh@mail.sysu.edu.cn

Address: No. 26, Yuancunerheng Road, Guangzhou 510655, China.

The contact person for reporting SAEs should fax the SAE report form signed by the investigator to the group leader within one working day after receiving the SAE report from the investigator. SAEs must be reported in accordance with the International Council for Harmonization (ICH) Guideline for Clinical Safety Data Management: Definitions and Standards for Expedited Reporting.

9.5.3 Reporting of non-SAEs

In addition to specific AEs, other non-SAEs will also be recorded by the investigator on the AE page in the CRF and will be recorded in a retrieval format. The following are the minimum requirements for recording in the AE reporting form: the identity of the subject, the drug, the duration of the AE, the start date of the event and the causality.

9.5.4 Follow-up of laboratory abnormalities

If there is an unexplainable abnormal value of the laboratory test, and the abnormality is clinically related, the test should be conducted again immediately and follow-up should be tracked until the test value returns to normal and/or a reasonable explanation for the abnormal situation is found. Any clear explanation should be recorded in the CRF.

10. Statistical methods

10.1 Trial objectives

10.1.1 Primary objective:

To evaluate the PK characteristics, assess safety, and determine the MTD of cetuximab in combination with capecitabine every three week on the basis of the occurrence of DLT.

10.1.2 Secondary objective:

To evaluate the efficacy parameters including PFS, OS, ORR, DCR and QOL of cetuximab in combination with capecitabine as first-line maintenance therapy in patients with RAS/BRAF wild-type mCRC.

10.1.3 Exploratory objective

Monitoring changes in genetic mutations during cetuximab treatment using circulating tumor DNA (ctDNA) analysis.

10.2 Definition of study endpoints

10.2.1 Primary endpoints

DLT was defined as any grade 3 or 4 haematological or non-haematological toxicity or administration of <66% of the assigned dose (i.e. a delay of >14 days in the first 6 weeks) due to toxicity at any of the cetuximab doses. If none of the patients treated at the starting dose level experienced a DLT, successive cohorts of three fully evaluable patients were enrolled at the next cetuximab dose levels. If one of three patients experiences a DLT, expand the cohort to six patients: If ≤1 of six patients have DLTs, escalate.If ≥2 of six have DLTs, halt escalation and declare the previous dose as MTD. If ≥2 of the initial three patients have DLTs, the MTD is exceeded, and the prior dose level is expanded to confirm MTD.

Safety assessed by evaluation of treatment-related adverse events. Adverse events were monitored and recorded from the time of informed consent until 90 days after the last dose of the investigational drug and were graded according to the National Cancer Institute Common Terminology Criteria for Adverse Events version 5.0.

Pharmacokinetic (PK) characteristics were assessed by measuring serum drug concentrations before and after each cycle of cetuximab administration. The evaluated parameters included: maximum serum concentration (C_{max}), trough plasma concentration (C_{min}), time to maximum concentration (T_{max}), average serum concentration at steady state (C_{av}), area under the plasma concentration-time curve from time zero to the last sampling time (AUC_{0-t}), apparent terminal elimination half-life associated with the negative terminal slope ($t_{1/2}$), total body clearance of drug from plasma (CL), trough concentrations before the second infusion and the volume of distribution at steady state (V_{ss}).

10.2.2 Secondary endpoints

PFS: It was defined as the time from initiation of maintenance treatment to tumor progression or death from any cause.

OS: It was defined as the time from initiation of maintenance treatment to death from any cause.

ORR: It was defined as the proportion of patients achieving a complete response (CR) or partial response (PR) as assessed by the investigator according to RECIST v1.1 criteria.

DCR: It was defined as the proportion of patients who achieved a complete response (CR), partial response (PR), or stable disease (SD) as their best overall response.

QOL: Quality of life was assessed at baseline, prior to initiation of cetuximab in combination with capecitabine, and again at 3 months after treatment initiation using the validated Chinese versions of the European Organisation for Research and Treatment of Cancer (EORTC) Quality of Life Questionnaire-Core 30 (QLQ-C30, version 3.0) and the colorectal cancer-specific module module QLQ-CR29. The QLQ-C30 is composed of 30 ordinal items assessing global health status/QOL (GHS/QOL), 5 functional domains and 9 symptoms. The QLQ-CR29 is includes 29 items assessing 4 functional domains and 18 symptoms. HRQOL scores were calculated according to the EORTC recommendations. Higher scores on functional and global health scales indicate better functioning,

while higher symptom scores indicate greater symptom burden.

10.2.3 Exploratory endpoint

The types and frequencies of secondary gene mutations were identified through analysis of circulating tumor DNA (ctDNA) at baseline, prior to initiation of cetuximab in combination with capecitabine, and again at the time of disease progression following treatment.

10.3 Calculation of sample size

This was a phase Ib dose-escalation study that employed a traditional 3+3 design. Based on a set of pre-specified rules and the inclusion of four dose-escalation cohorts, the estimated sample size ranged from a minimum of 3 patients to a maximum of 24 patients (up to 6 patients per cohort).

To further evaluate the pharmacokinetic and efficacy comparability between the dose-escalation regimens and the standard dosing regimen of cetuximab at 500 mg/m^2 every two weeks, an additional six patients were enrolled to receive cetuximab at 500 mg/m^2 in combination with 5-fluorouracil (5-FU) administered biweekly.

10.4 Analysis populations

10.4.1 Safety analysis set

All safety analyses were conducted in patients who received at least one dose of the study medication and had available safety data. Safety outcomes were summarized as the frequency and percentage of patients who experienced each adverse event.

10.4.2 PK analyses set

All enrolled subjects who received the investigational drug and had at least one valid plasma concentration measurement during the study were included in the PK analysis set.

10.4.3 Efficacy analyses set

All enrolled subjects who received the investigational drug were included in the efficacy analysis set.

11. Ethical considerations

Investigators must ensure that the trial is conducted in full compliance with the principles of the ‘Declaration of Helsinki’ or the laws and regulations of the place where the trial is conducted to achieve the maximum protection of individuals. The trial must fully comply with the principles listed in the ICH-GCP Guidelines (January 1997), or comply with the requirements of local regulations to provide greater protection to the subjects. In other countries with GCP, investigators are required to strictly abide by the relevant provisions.

12. References

1. Bray F, Ferlay J, Soerjomataram I, Siegel RL, Torre LA, Jemal A. Global cancer statistics 2018: GLOBOCAN estimates of incidence and mortality worldwide for 36 cancers in 185 countries. *CA Cancer J Clin*. 2018;68(6):394-424. doi:10.3322/caac.21492.

2. Benson AB, Venook AP, Al-Hawary MM, et al. Anal Carcinoma, Version 2.2018, NCCN Clinical Practice Guidelines in Oncology. *J Natl Compr Canc Netw*. 2018;16(7):852-871. doi:10.6004/jnccn.2018.0060.

3. Heinemann V, von Weikersthal LF, Decker T, et al. FOLFIRI plus cetuximab versus FOLFIRI plus bevacizumab as first-line treatment for patients with metastatic colorectal cancer (FIRE-3): a randomised, open-label, phase 3 trial. *Lancet Oncol*. 2014;15(10):1065-1075. doi:10.1016/S1470-2045(14)70330-4.

4. Ye LC, Liu TS, Ren L, et al. Randomized controlled trial of cetuximab plus chemotherapy for patients with KRAS wild-type unresectable colorectal liver-limited metastases. *J Clin Oncol*. 2013;31(16):1931-1938. doi:10.1200/JCO.2012.44.8308.

5. Bokemeyer C, Bondarenko I, Makhson A, et al. Fluorouracil, leucovorin, and oxaliplatin with and without cetuximab in the first-line treatment of metastatic colorectal cancer. *J Clin Oncol*. 2009;27(5):663-671. doi:10.1200/JCO.2008.20.8397.
6. Lenz HJ, Ou FS, Venook AP, et al. Impact of Consensus Molecular Subtype on Survival in Patients With Metastatic Colorectal Cancer: Results From CALGB/SWOG 80405 (Alliance). *J Clin Oncol*. 2019;37(22):1876-1885. doi:10.1200/JCO.18.02258.
7. Tournigand C, Cervantes A, Figer A, et al. OPTIMOX1: a randomized study of FOLFOX4 or FOLFOX7 with oxaliplatin in a stop-and-Go fashion in advanced colorectal cancer--a GERCOR study. *J Clin Oncol*. 2006;24(3):394-400. doi:10.1200/JCO.2005.03.0106.
8. Chibaudel B, Maindrault-Goebel F, Lledo G, et al. Can chemotherapy be discontinued in unresectable metastatic colorectal cancer? The GERCOR OPTIMOX2 Study. *J Clin Oncol*. 2009;27(34):5727-5733. doi:10.1200/JCO.2009.23.4344.
9. Yalcin S, Uslu R, Dane F, et al. Bevacizumab + capecitabine as maintenance therapy after initial bevacizumab + XELOX treatment in previously untreated patients with metastatic colorectal cancer: phase III 'Stop and Go' study results--a Turkish Oncology Group Trial. *Oncology*. 2013;85(6):328-335. doi:10.1159/000355914.
10. Simkens LH, van Tinteren H, May A, et al. Maintenance treatment with capecitabine and bevacizumab in metastatic colorectal cancer (CAIRO3): a phase 3 randomised controlled trial of the Dutch Colorectal Cancer Group. *Lancet*. 2015;385(9980):1843-1852. doi:10.1016/S0140-6736(14)62004-3.
11. Wasan H, Meade AM, Adams R, et al. Intermittent chemotherapy plus either intermittent or continuous cetuximab for first-line treatment of patients with KRAS wild-type advanced colorectal cancer (COIN-B): a randomised phase 2 trial. *Lancet Oncol*. 2014;15(6):631-639. doi:10.1016/S1470-2045(14)70106-8.
12. Aranda E, García-Alfonso P, Benavides M, et al. First-line mFOLFOX plus cetuximab followed by mFOLFOX plus cetuximab or single-agent cetuximab as maintenance therapy in patients with metastatic colorectal cancer: Phase II randomised MACRO2 TTD study. *Eur J Cancer*. 2018;101:263-272. doi:10.1016/j.ejca.2018.06.024.
13. Tveit KM, Guren T, Glimelius B, et al. Phase III trial of cetuximab with continuous or intermittent fluorouracil, leucovorin, and oxaliplatin (Nordic FLOX) versus FLOX alone in first-line treatment of metastatic colorectal cancer: the NORDIC-VII study. *J Clin Oncol*. 2012;30(15):1755-1762. doi:10.1200/JCO.2011.38.0915.
14. Boige V, Francois E, Blons H, et al. 387P - Maintenance treatment with cetuximab versus observation in RAS wild-type metastatic colorectal cancer: final results of the randomized phase II TIME-PRODIGE 28 UNICANCER stud[J]. *Ann Oncol*. 2022, 33 suppl 7:S713-S714. DOI: 10.1016/j.annonc.2022.07.526.
15. Cremolini C, Antoniotti C, Lonardi S, et al. Activity and Safety of Cetuximab Plus Modified FOLFOXIRI Followed by Maintenance With Cetuximab or Bevacizumab for RAS and BRAF Wild-type Metastatic Colorectal Cancer: A Randomized Phase 2 Clinical Trial. *JAMA Oncol*. 2018;4(4):529-536. doi:10.1001/jamaoncol.2017.5314.
16. Pietrantonio F, Morano F, Corallo S, et al. Maintenance Therapy With Panitumumab Alone vs Panitumumab Plus Fluorouracil-Leucovorin in Patients With RAS Wild-Type Metastatic Colorectal Cancer: A Phase 2 Randomized Clinical Trial. *JAMA Oncol*. 2019;5(9):1268-1275. doi:10.1001/jamaoncol.2019.1467.

17. Modest DP, Karthaus M, Fruehauf S, et al. Panitumumab Plus Fluorouracil and Folinic Acid Versus Fluorouracil and Folinic Acid Alone as Maintenance Therapy in *RAS* Wild-Type Metastatic Colorectal Cancer: The Randomized PANAMA Trial (AIO KRK 0212). *J Clin Oncol*. 2022;40(1):72-82. doi:10.1200/JCO.21.01332.
18. Wang L, Liu Y, Yin X, et al. Effect of Reduced-Dose Capecitabine Plus Cetuximab as Maintenance Therapy for RAS Wild-Type Metastatic Colorectal Cancer: A Phase 2 Clinical Trial. *JAMA Netw Open*. 2020;3(7):e2011036. Published 2020 Jul 1. doi:10.1001/jamanetworkopen.2020.11036.
19. Jiang T, Chen H, Zheng J, et al. Cetuximab Maintenance Therapy in Patients with Unresectable Wild-Type RAS and BRAF Metastatic Colorectal Cancer: A Single-Institute Prospective Study. *Adv Ther*. 2020;37(6):2829-2840. doi:10.1007/s12325-020-01360-8.

Supplementary Appendix: CONSORT 2025 checklist

Section / Topic	No	CONSORT 2025 checklist item description	Reported on page no.
**Title and abstract**			
Title and structured abstract	1a	Identification as a randomised trial	N/A, as this was a non-randomized exploratory phase Ib study
	1b	Structured summary of the trial design, methods, results, and conclusions	2
**Open science**			
Trial registration	2	Name of trial registry, identifying number (with URL) and date of registration	2, 11
Protocol and statistical analysis plan	3	Where the trial protocol and statistical analysis plan can be accessed	14-17, Supplement page 13-43
Data sharing	4	Where and how the individual de-identified participant data (including data dictionary), statistical code and any other materials can be accessed	18
Funding and conflicts of interest	5a	Sources of funding and other support (e.g., supply of drugs), and role of funders in the design, conduct, analysis and reporting of the trial	18
	5b	Financial and other conflicts of interest of the manuscript authors	18
**Introduction**			
Background and rationale	6	Scientific background and rationale	3-4
Objectives	7	Specific objectives related to benefits and harms	4, Supplement page 28-29
**Methods**			

Patient and public involvement	8	Details of patient or public involvement in the design, conduct and reporting of the trial	11
Trial design	9	Description of trial design including type of trial (e.g., parallel group, crossover), allocation ratio, and framework (e.g., superiority, equivalence, non-inferiority, exploratory)	11-12
Changes to trial protocol	10	Important changes to the trial after it commenced including any outcomes or analyses that were not prespecified, with reason	N/A
Trial setting	11	Settings (e.g., community, hospital) and locations (e.g., countries, sites) where the trial was conducted	11-12
Eligibility criteria	12a	Eligibility criteria for participants	12
	12b	If applicable, eligibility criteria for sites and for individuals delivering the interventions (e.g., surgeons, physiotherapists)	13
Intervention and comparator	13	Intervention and comparator with sufficient details to allow replication. If relevant, where additional materials describing the intervention and comparator (e.g., intervention manual) can be accessed	13
Outcomes	14	Pre-specified primary and secondary outcomes, including the specific measurement variable (e.g., systolic blood pressure), analysis metric (e.g., change from baseline, final value, time to event), method of aggregation (e.g., median, proportion), and time point for each outcome	13-14
Harms	15	How harms were defined and assessed (e.g., systematically, non-systematically)	15-16
Sample size	16a	How sample size was determined, including all assumptions supporting the sample size calculation	12
	16b	Explanation of any interim analyses and stopping guidelines	12, 16
Randomisation:			
Sequence generation	17a	Who generated the random allocation sequence and the method used	N/A
	17b	Type of randomisation and details of any restriction (e.g., stratification, blocking and block size)	N/A
Allocation concealment mechanism	18	Mechanism used to implement the random allocation sequence (e.g., central computer/telephone; sequentially numbered, opaque, sealed containers), describing any steps to conceal the sequence until interventions were assigned	N/A
Implementation	19	Whether the personnel who enrolled and those who assigned participants to the interventions had access to the	N/A

		random allocation sequence	
Blinding	20a	Who was blinded after assignment to interventions (e.g., participants, care providers, outcome assessors, data analysts)	N/A
	20b	If blinded, how blinding was achieved and description of the similarity of interventions	N/A
Statistical methods	21a	Statistical methods used to compare groups for primary and secondary outcomes, including harms	14-17
	21b	Definition of who is included in each analysis (e.g., all randomised participants), and in which group	16-17
	21c	How missing data were handled in the analysis	17
	21d	Methods for any additional analyses (e.g., subgroup and sensitivity analyses), distinguishing prespecified from post-hoc	17
**Results**			
Participant flow, including flow diagram	22a	For each group, the numbers of participants who were randomly assigned, received intended intervention, and were analysed for the primary outcome	4
	22b	For each group, losses and exclusions after randomisation, together with reasons	N/A
Recruitment	23a	Dates defining the periods of recruitment and follow-up for outcomes of benefits and harms	4, 7, 16
	23b	If relevant, why the trial ended or was stopped	N/A
Intervention and comparator delivery	24a	Intervention and comparator as they were actually administered (e.g., where appropriate, who delivered the intervention/comparator, how participants adhered, whether they were delivered as intended [fidelity])	4-5
	24b	Concomitant care received during the trial for each group	5
Baseline data	25	A table showing baseline demographic and clinical characteristics for each group	4, Supplement page 2-3
Numbers analysed, outcomes and estimation	26	For each primary and secondary outcome, by group:<br>● the number of participants included in the analysis<br>● the number of participants with available data at the outcome time point<br>● result for each group, and the estimated effect size and its precision (such as 95% confidence interval)	4-7

		• for binary outcomes, presentation of both absolute and relative effect size	
Harms	27	All harms or unintended events in each group	4-5
Ancillary analyses	28	Any other analyses performed, including subgroup and sensitivity analyses, distinguishing pre-specified from post-hoc	N/A
**Discussion**			
Interpretation	29	Interpretation consistent with results, balancing benefits and harms, and considering other relevant evidence	8-10
Limitations	30	Trial limitations, addressing sources of potential bias, imprecision, generalisability, and, if relevant, multiplicity of analyses	10-11
